# Supplementary material for: New Viral Sequences Identified in the Flavescence Dorée Phytoplasma Vector Scaphoideus titanus
Source: Viruses. 2020 Mar 6;12(3):287. doi: 10.3390/v12030287 (PMC7150801; doi:10.3390/v12030287)
Supplement: Supplementary file 1 [file viruses-12-00287-s001.zip › Supplementary _Materials/Supplementary_materialS4.pdf]

S4

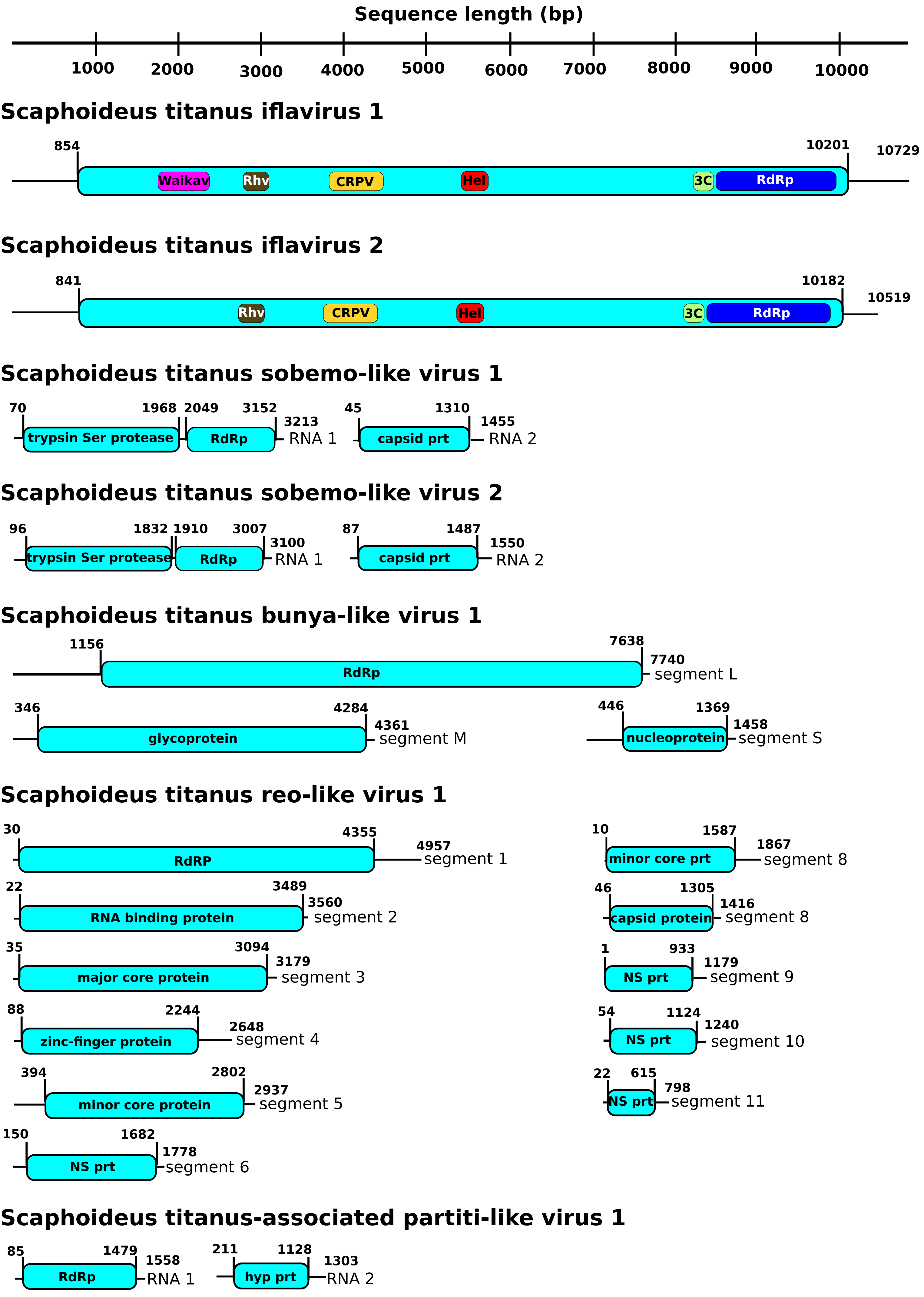
Sequence length (bp)
1000
2000
3000
4000
5000
6000
7000
8000
9000
10000
Scaphoideus titanus iflavirus 1
854
10201
10729
Waikav
Rhv
CRPV
Hel
3C
RdRp
Scaphoideus titanus iflavirus 2
841
10182
10519
Rhv
CRPV
Hel
3C
RdRp
Scaphoideus titanus sobemo-like virus 1
70
1968
2049
3152
3213
trypsin Ser protease
RdRp
RNA 1
45
1310
1455
capsid prt
RNA 2
Scaphoideus titanus sobemo-like virus 2
96
1832
1910
3007
3100
trypsin Ser protease
RdRp
RNA 1
87
1487
1550
capsid prt
RNA 2
Scaphoideus titanus bunya-like virus 1
1156
7638
7740
RdRp
segment L
346
4284
4361
glycoprotein
segment M
446
1369
1458
nucleoprotein
segment S
Scaphoideus titanus reo-like virus 1
30
4355
4957
RdRP
segment 1
10
1587
1867
minor core prt
segment 8
22
3489
3560
RNA binding protein
segment 2
46
1305
1416
capsid protein
segment 8
35
3094
3179
major core protein
segment 3
1
933
1179
NS prt
segment 9
88
2244
2648
zinc-finger protein
segment 4
54
1124
1240
NS prt
segment 10
394
2802
2937
minor core protein
segment 5
22
615
798
NS prt
segment 11
150
1682
1778
NS prt
segment 6
Scaphoideus titanus-associated partiti-like virus 1
85
1479
1558
RdRp
RNA 1
211
1128
1303
hyp prt
RNA 2
