# Supplementary figures and images for: New Viral Sequences Identified in the Flavescence Dorée Phytoplasma Vector Scaphoideus titanus
Source: Viruses. 2020 Mar 6;12(3):287. doi: 10.3390/v12030287 (PMC7150801; doi:10.3390/v12030287)

# S1

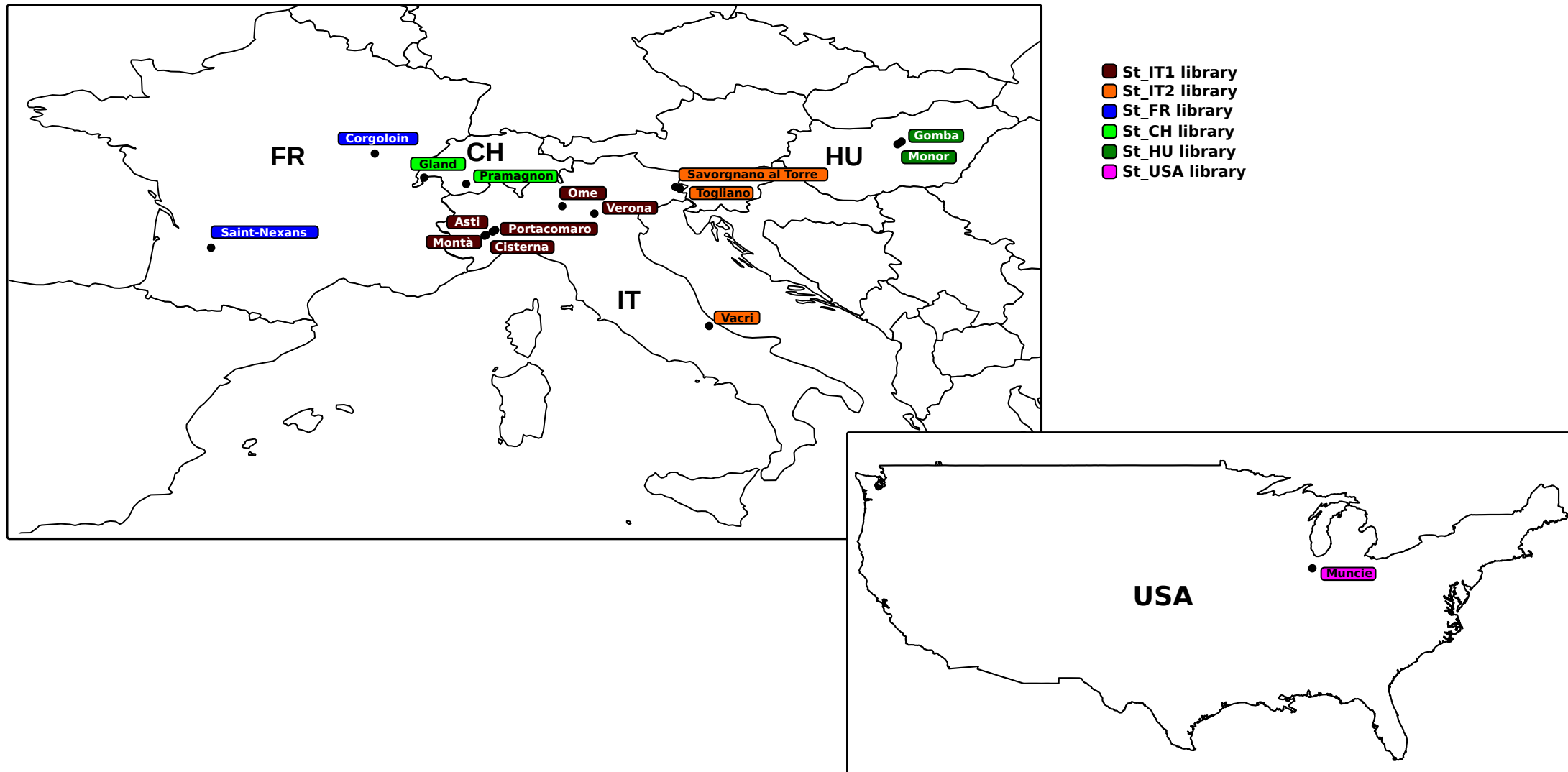

Supplement: Supplementary file 1 [file viruses-12-00287-s001.zip › Supplementary _Materials/Supplementary_materialS1.pdf]

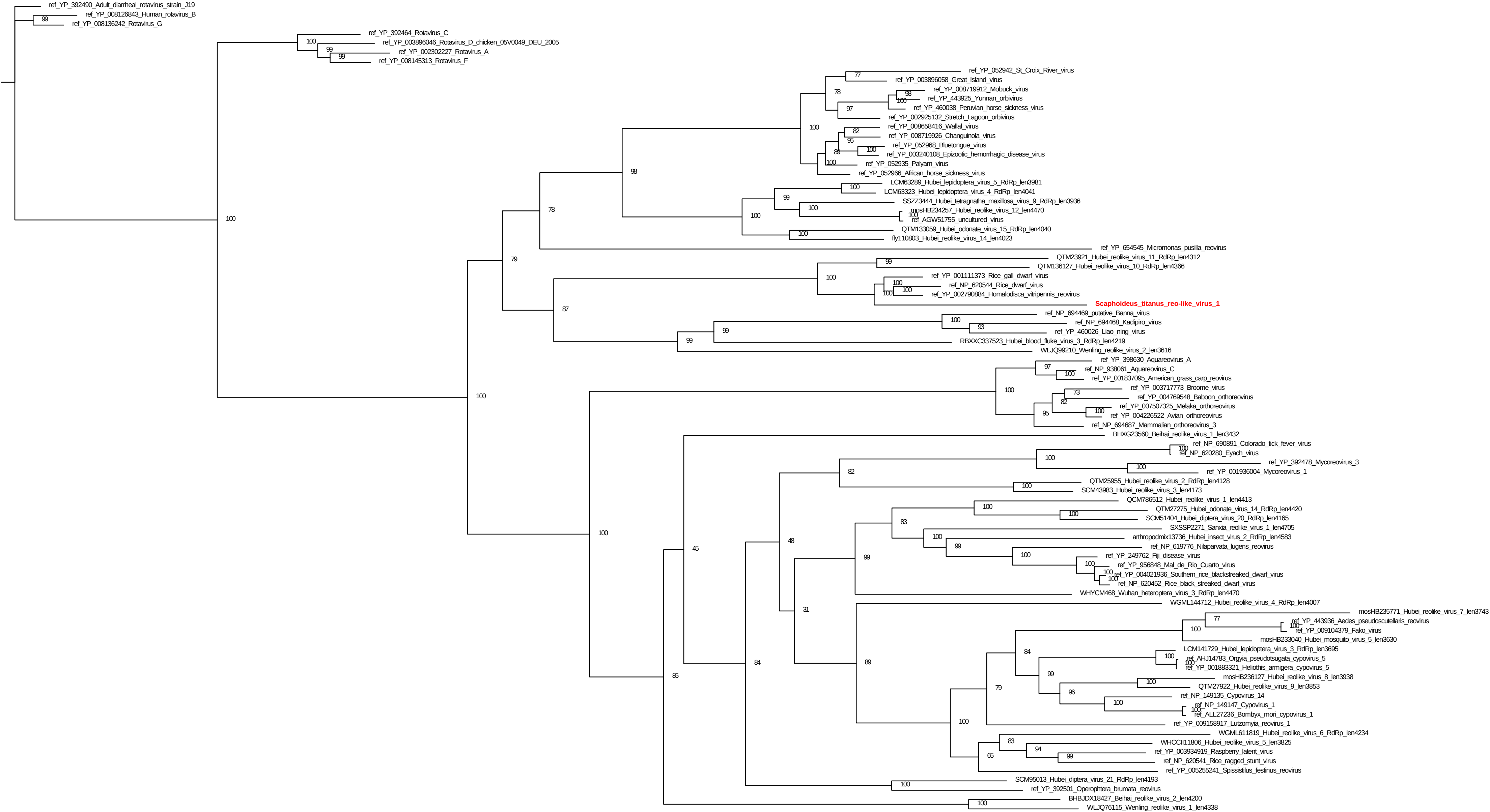

Supplement: Supplementary file 1 [file viruses-12-00287-s001.zip › Supplementary _Materials/Supplementary_materialS10.pdf]

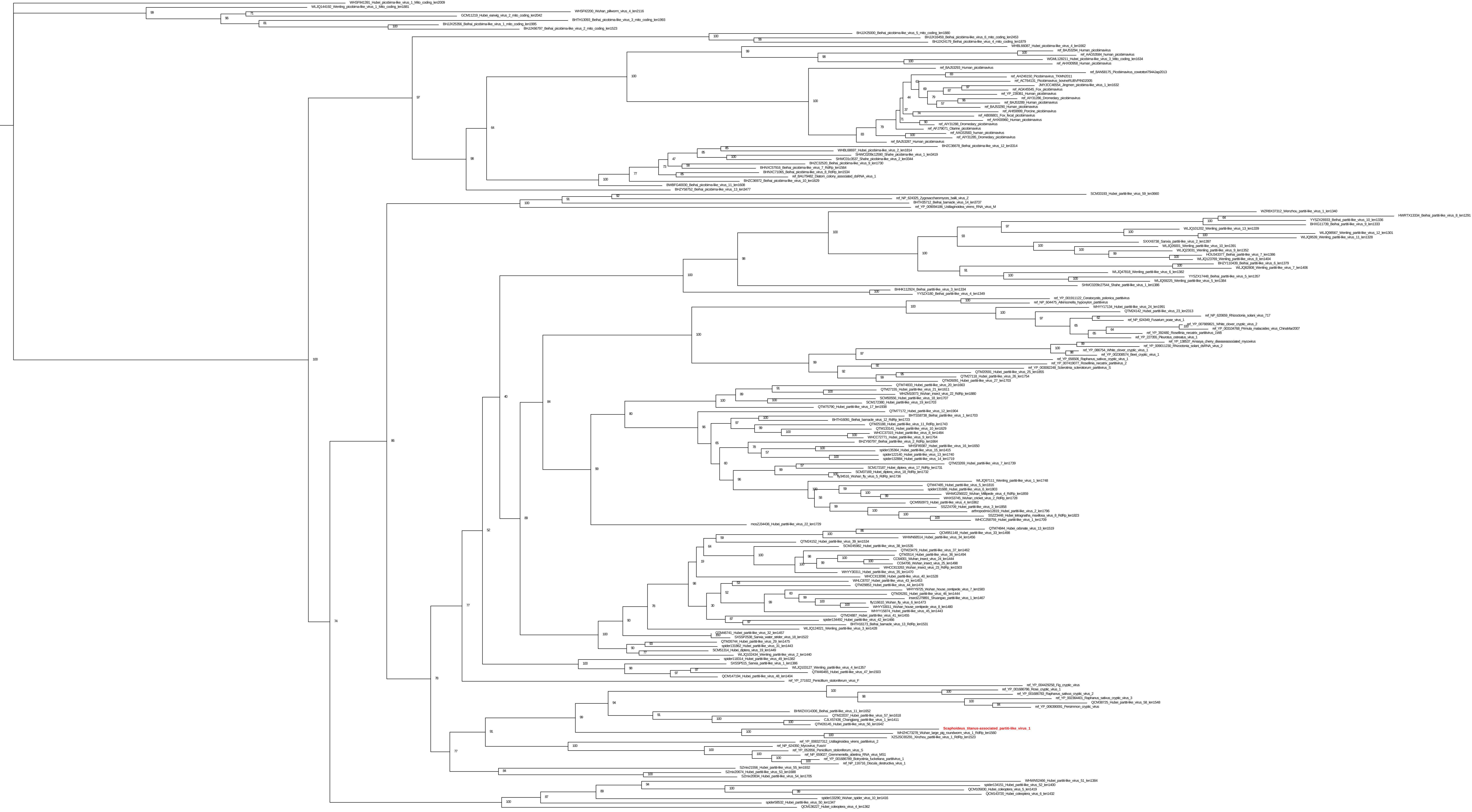

Supplement: Supplementary file 1 [file viruses-12-00287-s001.zip › Supplementary _Materials/Supplementary_materialS11.pdf]

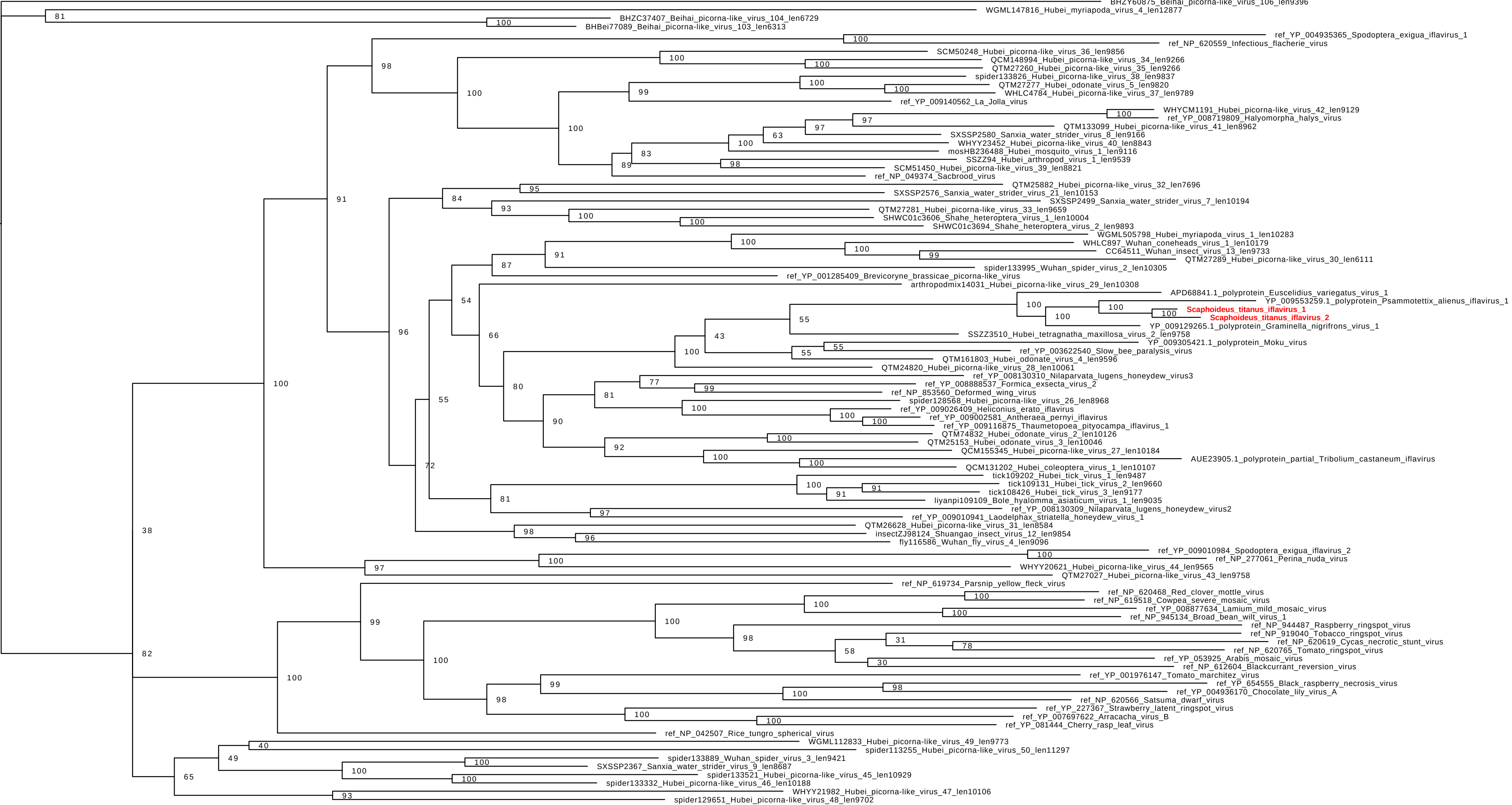

Supplement: Supplementary file 1 [file viruses-12-00287-s001.zip › Supplementary _Materials/Supplementary_materialS5.pdf]

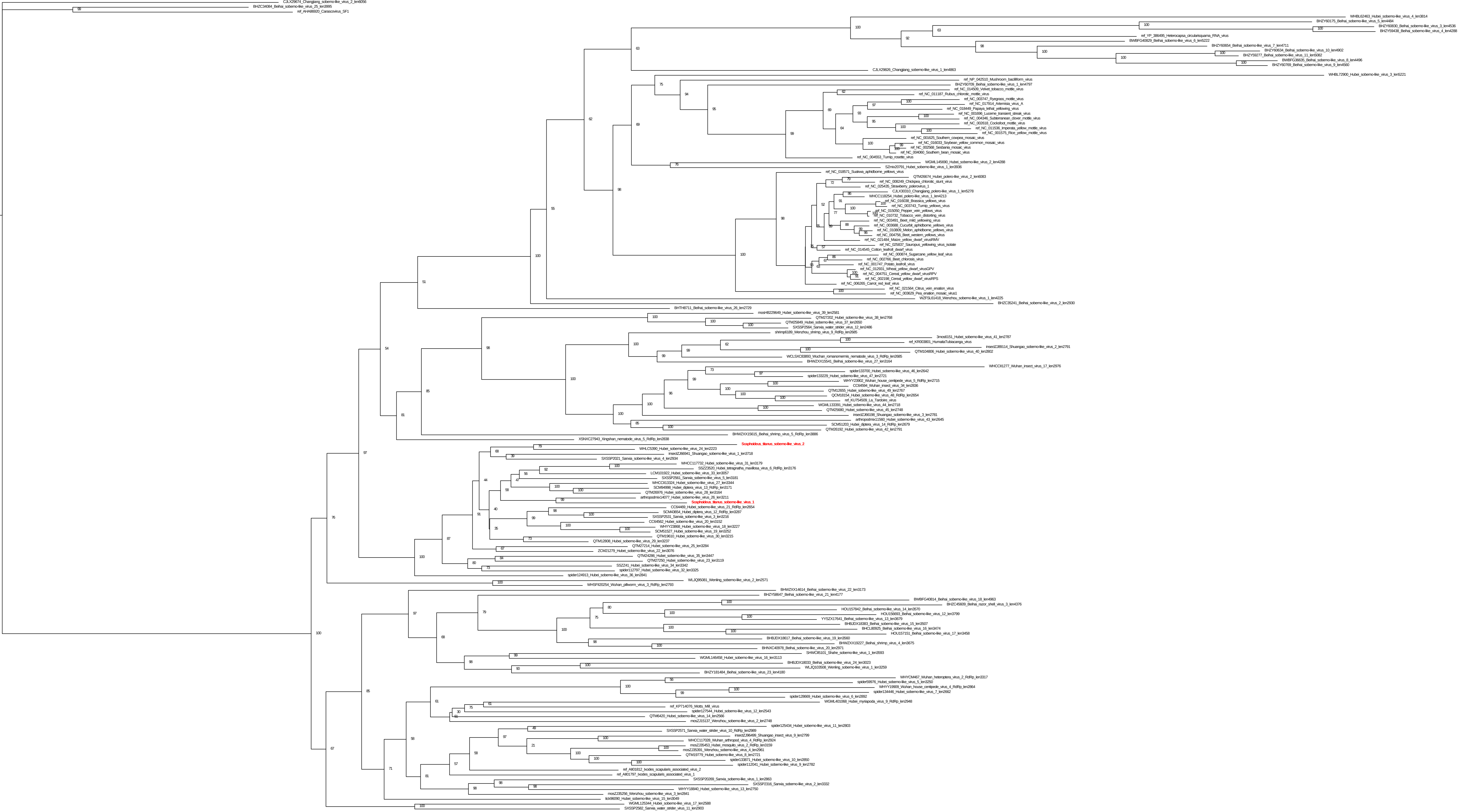

Supplement: Supplementary file 1 [file viruses-12-00287-s001.zip › Supplementary _Materials/Supplementary_materialS6.pdf]

**S7**

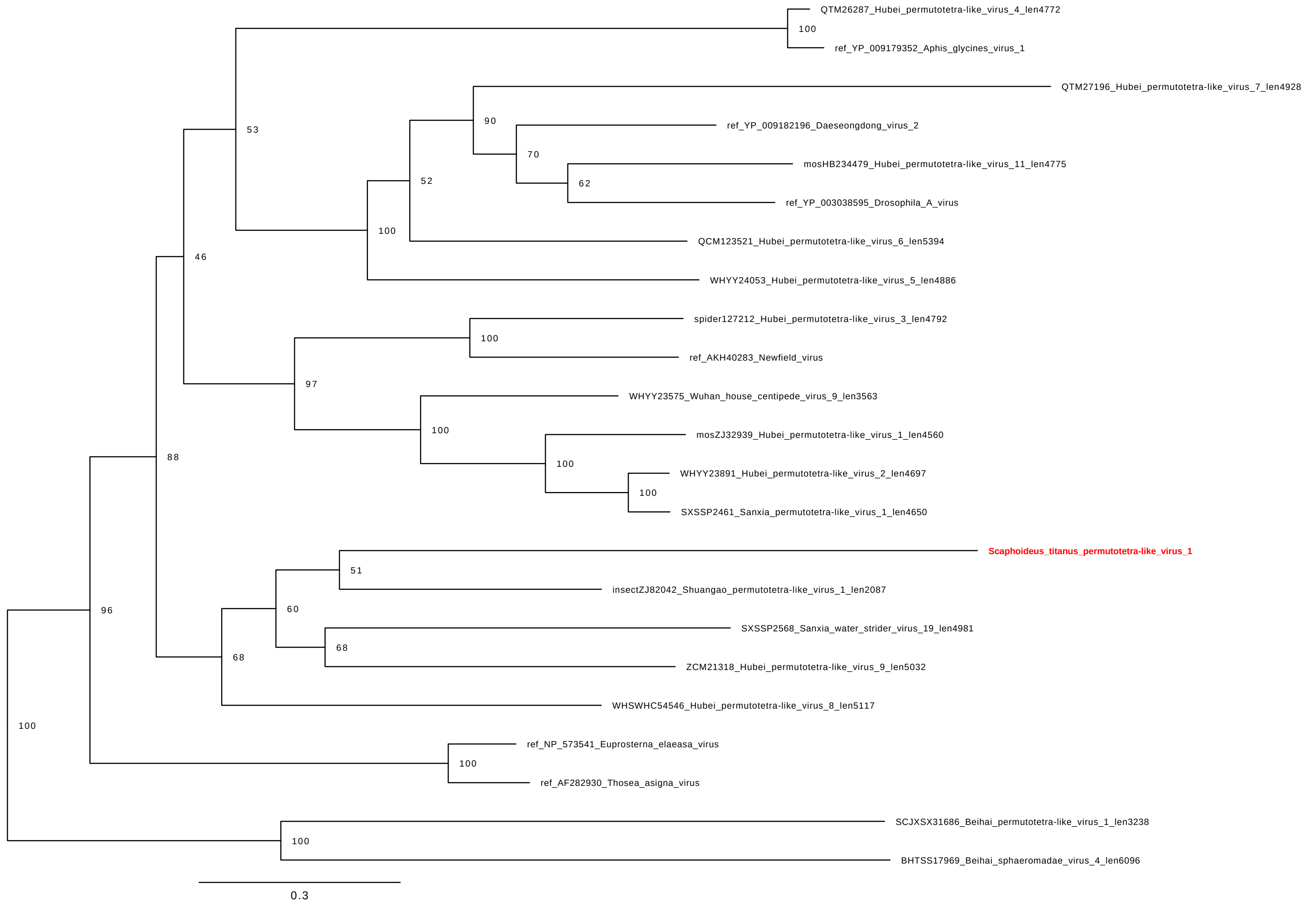

Supplement: Supplementary file 1 [file viruses-12-00287-s001.zip › Supplementary _Materials/Supplementary_materialS7.pdf]

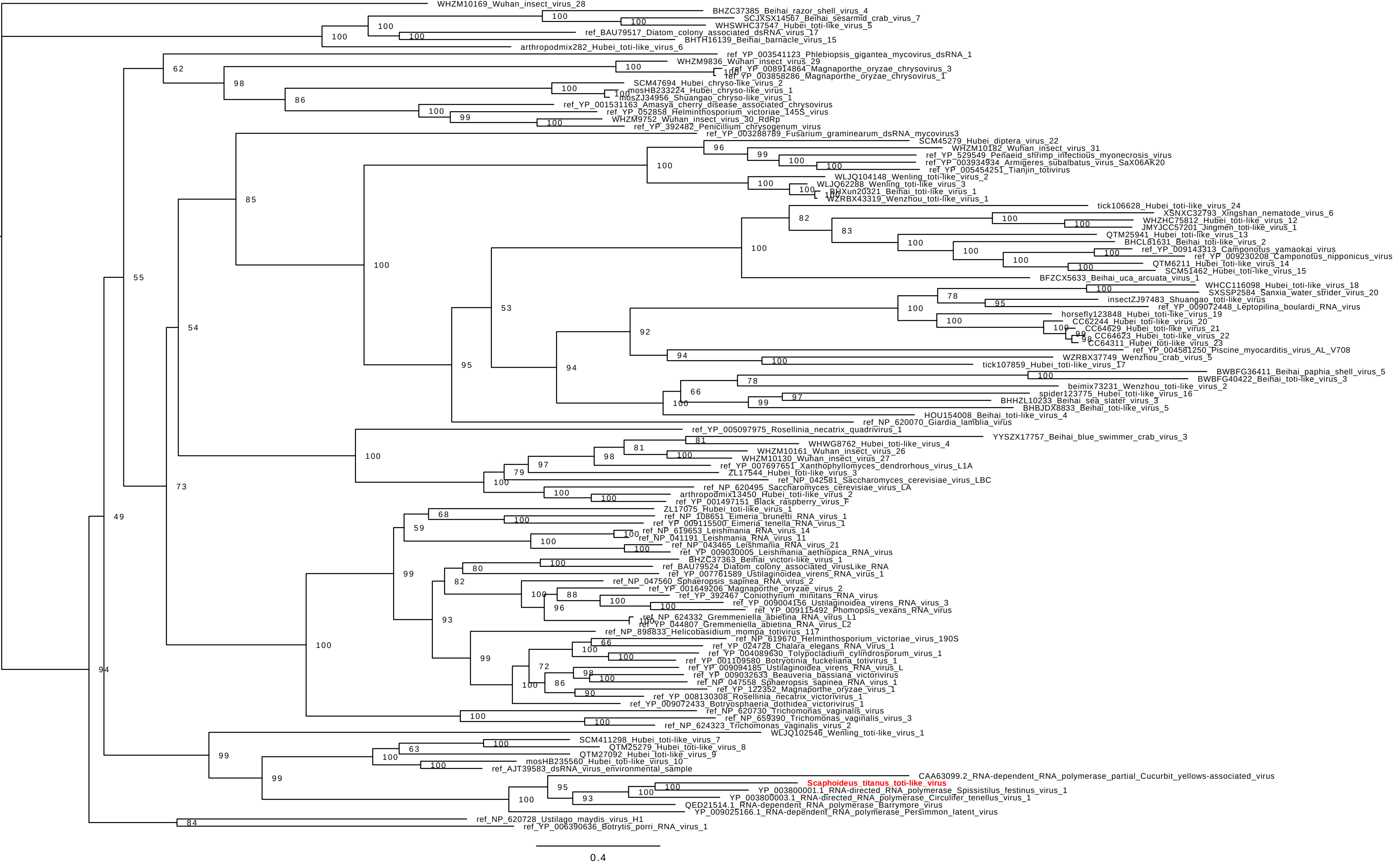

Supplement: Supplementary file 1 [file viruses-12-00287-s001.zip › Supplementary _Materials/Supplementary_materialS9.pdf]
