# Supplementary material for: New Viral Sequences Identified in the Flavescence Dorée Phytoplasma Vector Scaphoideus titanus
Source: Viruses. 2020 Mar 6;12(3):287. doi: 10.3390/v12030287 (PMC7150801; doi:10.3390/v12030287)

S8

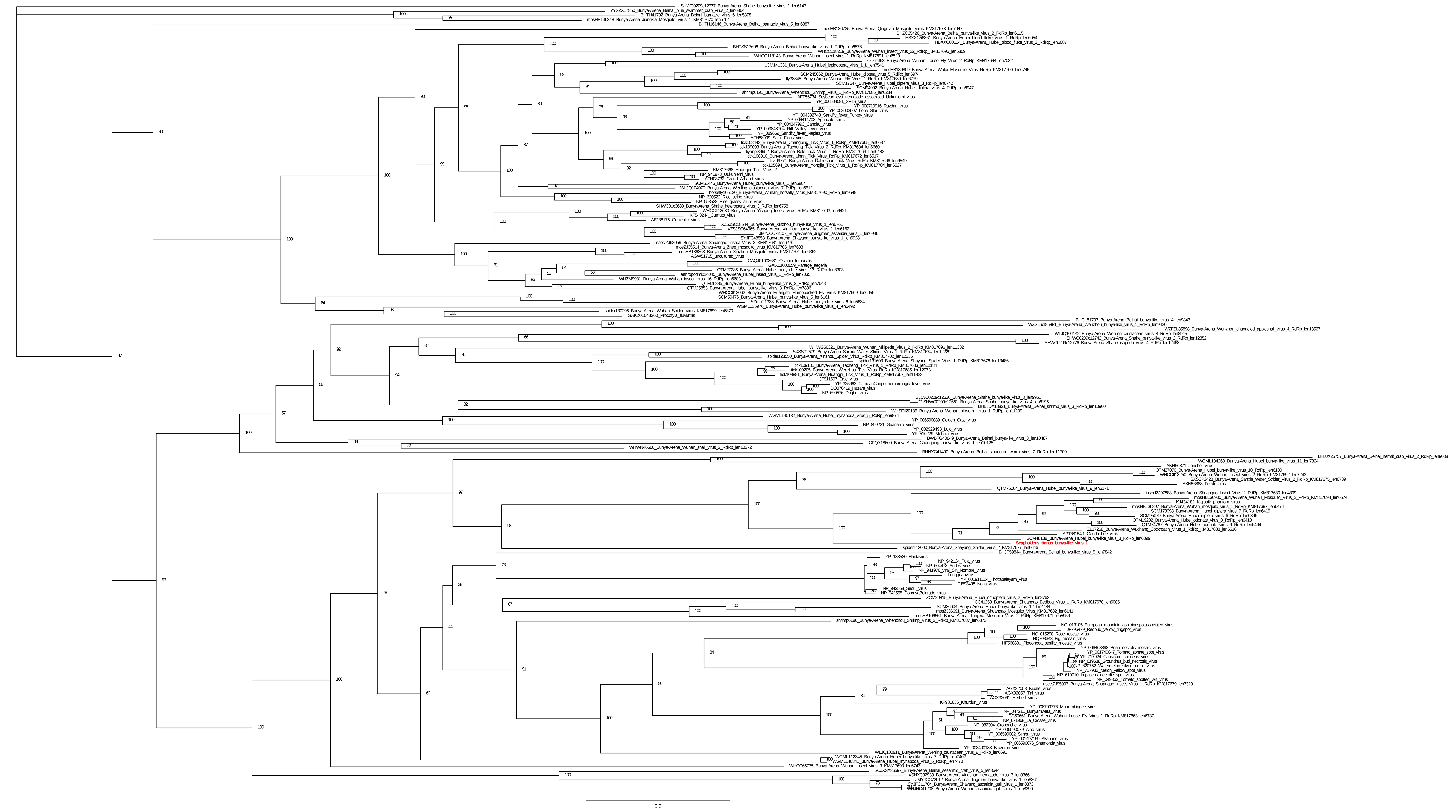

Supplement: Supplementary file 1 [file Supplementary_materialS8.pdf › Supplementary _Materials/Supplementary_materialS8.pdf]
