# Supplementary material for: New Viral Sequences Identified in the Flavescence Dorée Phytoplasma Vector Scaphoideus titanus
Source: Viruses. 2020 Mar 6;12(3):287. doi: 10.3390/v12030287 (PMC7150801; doi:10.3390/v12030287)
Supplement: Supplementary file 1 [file viruses-12-00287-s001.zip › Supplementary _Materials/Supplementary_materialS12.doc]

**Supplementary material S12** Sequences of potential endogenous viral elements (EVEs) found in the European RNA-Seq libraries. Each sequence header reports the internal code assigned to the sequence, the best blastx hit and the library code the sequence was identified from. Binding positions of the primers used in PCR to demonstrate the integration into *S. titanus* genome (see Supplementary material S3) are highlighted in yellow.

**Mononegavirus 1**

>TRINITY_DN91784_c0_g1 YP_009288955.1 RNA-dependent RNA polymerase [Sanxia water strider virus 4] from the St_IT2 library

AGAAGATCACAAATGGTATCGGAATGGATTTGAAATTACAAGAATCCTGGGTCTCGACAACATTATTAAACTACGGAAAGAAGATAATAGTGGATGGGAGCTTCACCAGCAGTGCTTTAAAGAAGATCAGGAGGGCGTATCAAGATGTAAGTGAATTGTATCCCAGTCTTCCGAACCGAGTATCTTCCGTATTTACCACTGCTCAATCCGCTTCTATGAAGTCATTTGACACTTTGTCTACCTACTATATAGCCCTGGTGAATTGTCTAAACATGATTGGGAAGAAAGGGAAGTACGGGACAACGATGGGAGGTCGCCTCAAGACGTTATTAGCTACAGAAGGAATCCAATTGGATGAGCATTTCAAACTGGTTTGTTTACTGCTCCCTAGAGAGTGTGGGGGGCTTCCAGTAATCCCTTACTTGAATATGCACTTCAGGGGGCACCCTGATCAGATAACATCTGTCCTTCCTTGGTTAAAAAAGCTGGGAGATAAGATCCCACAAGCAAAACGAATACTAGATCTAATAAACTCTGGTTACTTTAATAGCGAGACTGTAGATTATCTTCAGCTAATCCAGGATCCCTACTCGTTGAATTTCAAAAAACCAGCCCAGTCTTCGAATCGGGTTAAACACGCCCTGATAGAAGTTATGAAAAACACATCTCGTAACACGGACGTAGTTACTACGATGGAATCTTGTAATGAGGACTCTTTGAGTAACATGGCGGAATATCTATCTCAAAGTGATCCTCTGTTTCCAAGACTGCTGAATGAGATCTTGGGATTGTCTCCAGATGGAAGTATGCAGAGTTTCTTATCTATTTTTAGAGATATGAAGACCATGAAACAGATGTTACGTCCATCTGCCGCTAATGATCTGGTGTATAAGCTGAGAGAAGGAGATCTGGAACTGATGAAGCATATCATATCTGTGTCAAGGATTGTCGAGGCGAGCCGGTGCCATCGGTGGATATCAAAATCTCTTAGGGTAGATCCTAATGATTGGCTAACTGAAAAACAAATGTGTATTACTAATCTAGCAAGAGAACTTAGACAAGTAACTTGGAGGAGAAAGGTAGAGGGAGTAAGTATACCACATCCGTGCGAGCAGTCATCTATTCATCTATCAACTGGAGATACGTGCTCATTTCAGGAATGCGAAGGAGATGAGATGATAGTGTACGTAGTTTTGGATGATAGGCAAAGACCTACTGAACCCATTCAATTTGATAAAGGACCACTCAGACACTATCTAGGATCTGTGACCTCTGAAAAAAGATCTGGTCCTGTATTGAATTTTCCAAGAACAGAAAAAGCAATTAGAGCCGCTCAGCAACTATTGCGAGTTCTCAACTGGCACAATAAAGCAAGACTCTGAGTTTAGCTATGGAAAACTACT>

TRINITY_DN103462_c0_g5 YP_009288955.1 RNA-dependent RNA polymerase [Sanxia water strider virus 4] from the St_IT1 library

GAGATAAACTCAAGCTCCTAAACTGAGAGAAGTCAGAGGAAGAGATATATTAGGTGTTAAGTGCTGCCCAATACATTGAAGATCATGAAGAAGAGCTGACTAAACGAGTAGAAGCTTACTTAGACAACCTAAAGAAGATGGTATCGGAATGGATTTGAAATTACAAGAATCCTGGGTCTCGACAACATTATTAAACTACGGAAAGAAGATAATAGTGGATGGGAGCTTCACCAGCAGTGCTTTAAAGAAGATCAGCAGGGCGTATCAAGATGTAAGTGAATTGTATCCCAGTCTTCCGAACCGAGTATCTTCCGTATTTACCACTGCTCAATCCGCTTCTATGAAGTCATTTGACACTTTGTCTACCTACTATATAGCCCTGGTGAATTGTCTAAACATGATTGGGAAGAAAGGGAAGTACGGGACAACGATGGGAGGTCGCCTCAAGACGTTATTAGCTACAGAAGGAATCCAATTGGATGAGCATTTCAAACTGGTTTGTTTACTGCTCCCTAGAGAGTGTGGGGGGCTTCCAGTAATCCCTTACTTGAATATGCACTTCAGGGGGCACCCTGATCAGATAACATCTGTCCTTCCTTGGTTAAAAAAGCTGGGAGATAAGATCCCACAAGCAAAACGAATACTAGATCTAATAAACTCTGGTTACTTTAATAGCGAGACTGTAGATTATCTTCAGCTAATCCAGGATCCCTACTCGTTGAATTTCAAAAAACCAGTCCAGTCTTCGAATCGGGTTAAACACGCCCTGATAGAAGTTATGAAAAAC

>TRINITY_DN102665_c2_g2 YP_009288955.1 RNA-dependent RNA polymerase [Sanxia water strider virus 4] from the St_CH library

CCACTGCTCAATCCGCTTCTATGAAGTCATTTGACACTTTGTCTACCTACTATATAGCCCTGGTGAATTGTCTAAACATGATTGGGAAGAAAGGGAAGTACGGGACAACGATGGGAGGTCGCCTCAAGACGTTATTAGCTACAGAAGGAATCCAATTGGATGAGCATTTCAAACTGGTTTGTTTACTGCTCCCTAGAGAGTGTGGGGGGCTTCCAGTAATCCCTTACTTGAATATGCACTTCAGGGGGCACCCTGATCAGATAACATCTGTCCTTCCTTGGTTAAAAAAGCTGGGAGATAAGATCCCACAAGCAAAACGAATACTAGATCTAATAAACTCTGGTTACTTTAATAGCGAGACTGTAGATTATCTTCAGCTAATCCAGGATCCCTACTCGTTGAATTTCAAAAAACCAGTCCAGTCTTCGAATCGGGATAAACACGCCCTGATAGAAGTTATGAAAAACACATCTCGTAACACGGACGTAGTTACTACGATGGAATCTTGTAATGAGGAGTCTTTGAGTAACATGGCGGAATATCTATCTCAAAGTGATCCTCTGTTTCCAAGAGTGCTGAATGAGATCTTGAGATTGTCCCCAGATGGAAGTATGCAGAGTTTCTTATCTATTTTTAGAGATATGAAGACCATGAAACAGATGTTACGTCCATCTGCCGCTAATGATCTGGTGTATAAGCTGAGAGAAGGAGATCTGGAACTGATGAAGCATATCATATCTGTGTCAAGGATTGTCGAGGCGAGCCGGTGCCATCGGTGGATATCAAAATCTCTTAGGGTAGATCCTAATGATTGGCTAACTGAAAAACAAATGTGTATTACTAATCTAGCAAGAGAACTTAGACAAGTAACTTGGAGGAGAAAGGTAGAGGGAGTAAGTATACCACATCCGTGCGAGCAGTCATCTATTCATCTATCAACTGGAGATACGTGCTCATTTCAGGAATGCGAAGGAGATGAGATGATAGTGTAC

**Mononegavirus 2**

>TRINITY_DN122164_c0_g1 YP_009288955.1 RNA-dependent RNA polymerase [Sanxia water strider virus 4] from the St_HU library

GACGGGACATACGCTGGGCGAGTGTTACGGCTGTTGTTAGAGGAGAAACAATCTTGTGTGACGCAGCTGTCAAAAGAGCTTAGGGATGTTACGTGGAGGAAGAATGTAGAAGGAGTTAGTATCCATCATCCTTGTGAACAGACATCTATCCACCTCTCTCGTGGGGATACCTGCTCTTTAGATGGGTGTCAAGGAGAAGAAATGGTTGTCTATGTAGTTCTCGATGATCGACAGAGACCAAAAGAGCCTATCCAGTTCGATAAAGGGCCTTTGAGGCATTACTTAGGCTCAGGAACTTCTGAAAAGAGATCGGGTCCAGTCCTCAATTTCCCGAGAACTGAGAAGGCCATCAGAGCAGCCCAGCAACTGTTGAGAGTCTTAAATTGGACTATCGATCGAGACGACCCCAAAAATACTCTCATAGCTTTCTTAGATGAGTTGATTAGATCTCGTTGTGATGCCAGTAAGGAGTTCCTATTACTAACATCTGGAGTTAATTACGGAGGATCAGTACAGCATCGTTTCTCTGATGTGACATCGAAGCATGAGAGCCGGCCAGGAATAAGAGTGAATAATCACTCAAGGATAATGATCTCCAGTGATCAACTTGGAAAATATGCAAGAGGAAAAGATAACTACTCCATAGTATTCCAGTCCTTGTATCTCCAGGTATTACACCTTATCAACACAGTGTTGATTTACAACCCAGAGATTCTGAAAGACGGAAACTTAGTCTTCCATCAACATTTAGTCTGTAACCGATGTTTGAGTATGTTAGATGAACCTCCAATTAGATGTCGAGCGGATGCGCCTATAGTCCCTAAATTGGTTAACTTCCCCCTTGTGTTTGCGAGTGTTGGCCAAAAATCAGAAATGATGCCTCAACACTGTATAGAGAAC

>TRINITY_DN100904_c0_g1 YP_009288955.1 RNA-dependent RNA polymerase [Sanxia water strider virus 4] from the St_FR libraryGGCGAGTGTTACGGCTGTTGTTAGAGGAGAAACAATCTTGTGTGACGCAGCTGTCAAAAGAGCTTAGGGATGTTACGTGGAGGAAGAATGTAGAAGGAGTTAGTATCCCTCATCCTTGTGAACAAACATCTATCCACCTCTCTCGTGGGGATACCTGCTCTTTAGATGGGTGTCAAGGAGAAGAAATGGTTGTCTATGTAGTTCTCGATGATCGACAGAGACCAAAAGAGCCTATCCAGTTCGATAAAGGGCCTTTGAGGCATTACTTAGGCTCAGGAACTTCTGAAAAGAGATCGGGTCCAGTCCTCAATTTCCCGAGAACTGAGAAGGCCATCAGAGCAGCCCAGCAACTGTTGAGAGTCTTAAATTGGACTATCGATCGAGACGACCCCAAAAATACTCTCATAGCTTTCTTAGATGAGTTGATTAGATCTCGTTGTGATGCCAGTAAGGAGTTCCTATTACTAACATCTGGAGTTAATTACGGAGGACCAGTACAGCATCGTTTCTCTGATGTGACATCGAAGCATGAGAGCCGGCCAG

**Mononegavirus 3**

>TRINITY_DN65599_c0_g2_i1 YP_009336924.1 RNA-dependent RNA polymerase [Hubei odonate virus 10] from the St_IT1 library

AGGTATTAGCGAACTCTTCCAAAGCCTTCCGGAATTGTCTGATCAGAAGAGAGACCTGCTAAGAAGGTACGACTCAATAGAAATAACACAAACAGTTAGAGAATGGTGTGTCTACCGAAATGTTTGGGAGGCAATGAGGGATATTATGAAAGACATGCAAATTACAGATGATTACCGATTTAAAGAAGTAGCTAAAGGGTTGACCACTGGATCCTTCTTTGTCAACCCGTACTTTATGATATACCAGGAAAATCAGTATGTGTGTTGGCTACTAGATTATAATCAGGTACTGATAATCTCTGACACAGTTGGATCTAGGTTTTTATCCTTGTTTTTCTGTTCGATCCACGAAAAGATTGCAATATCACCGATGCCAAAAGTCTCTGAGATGTTGGAGTTCTATGTGACGGGGGATAACATATTGACTAAGTTTGGTAATTCTGGATACAACCATCTAAAACTCTTGGAATCTCTATCTATTGCAAGCTATCTATCCAGGTTTGAACCTCTAAAAATTAGTAAAAACTACTATCACCAACTACTTGAAGAAGCTGGAGATGATAAGGACCTACTAGAGAGTATGAAAATTTTATATGAAATCTTAACTCGAATAGGAGATCATCC

>TRINITY_DN98841_c0_g1 YP_009336924.1 RNA-dependent RNA polymerase [Hubei odonate virus 10] from the St_IT2 library

CGACTCAATAGAAATAACACAAACAGTTAGAGAATGGTGTGTCTACCGAAATGTTTGGGAGGCAATGAGGGATATTATGAAAGACATGCAAATTACAGATGATTACCGATTTAAAGAAGTAGCTAAAGGGTTGACCACTGGATTCTTCTTTGTCAACCCGTACTTTATGATATACCAGGAAAATCAGTATGTGTGTTGGCTACTAGATTATAATCAGGTACTGATAATCTCTGACACAGTTGGATCTAGGTTTTTATCCTTGTTTTTCTGTTCGATCCACGAAAAGATTGCAATATCACCGATGCCAAAAGTCTCTGAGATGTTGGAGTTCTATGTGACGGGGGATAACATATTGACTAAGTTTGGTAATTCTGGATACAACCATCTAAAACTCTTGGAATCTCTATCTATTGCAAGCTATCTATCCAGGTTTGAACCTCTAAAAATTAGTAAAAACTACTATCACCAACTACTTGAAGAAGCTGGAGATGATAAGGACCTACTAGAGAGTATGAAAATTTTATATGAAATCTTAACTCGAATAGGAGATCATCCCCTAAAGTTGTTTGAAGTTTATGGGTGTTTTAGACACTTTGGCCATCCTACTGTCGATGAAATAGAAGGAATCAAAAGTTTGAAAGAAAATAGTAGAATGGAGATAAAGATGGATCAAGAACAGCTACGAAAGGTTGGGAGGGCTTTTAATCGAATGATTATTCTAGAGTTTATCTCCACCAGAAAGAGGTGGGCCTAAGTGTCACCTCTCGGCACAGTGCCAGAATAGACAAATGAAAAATCTAGTTAAGAATCAGCCTGTGGCTATATCAGAGTATGATTTGGTCATCTCTCTTAATGACTGGTCATACATTGAATTTGATCAGGAGTTCCAATTCGATGATTTCCCAGACTTCACGTCTCTATTCTCAGATACTGCTATATCCCCATATCTGAGCCACTGGTACTCTGTATTTAGCAAAGACTTGTTGAATGTTTCGCATCCGACGGATATGGAAGAATCCCGTAGAGTTCTGATAGAAGTCCTTAAACGTGAAAAAATCAATTGTAAAGAGATCAGAGAAATAATTCAATGGCACAGAGTTTGCCAGTCAAAACCTAATAAATAATGAATTGCAGGCACAGAAGGTGCTTAACTGTTTTAATTATAAGTTGGAATAAACCCTGTAATTAATCCTTGCCTCTGGCCTAAGTCCAGATGGCACTCAAACCTTTAACCTTGCTTAAACAAAAGAAATATTCAATAATATTTACCCATTAAGCTTGAGCGGCAAATTAAATTGACAGAGATGACCCGGAAAACACTATGGTTTCTTTTGTCGAAGAGTTAATTCGGTCTCGTTGTGACACTAGCAAAGAATTCTTACTGCTCACATCTGGGGTGAACTATGGCGGATCTGTACAGAACCGTTTCTCAAATGTTACATCTAAACATGAGAGTCGCCCAAGAATACGAGTCAATAATCACTCTAGGGTCATGATCTCAAGTGATCAGTTAGGAAAGTATGCTAGAGGAAAGGACAATTACTCAATAGTGTTTCAATCTTTATATCTCCAAGTGCTTCATCTTATTAATACCTTGCTAGTTTATGACCCACACATCTTGAAAGATGGAAATTTAGTTTTACACCAACACTTAGTTTGTAAGGGGTGTCTAGACCTACTTGATGAACCTCCTATCTTAAATAAATCTTGTGCTCCTGAAGTCCCCAAGCTAATGAATTGTCCACTCATATTTGCCAGTATAGGACAAAAATCAGAGGCCCTACCTCAACATTGCATAGAGAATACTCCTACTGTGGAGCCTAACCCAAGAGACCCAGATATTAGGAGAAAGAGTGCTTACGCAGCTGCTCTTCTAATCATAGGAGAGACAAGTCATCTGGTCACACCTCTGCTCCATTCTAATATGAAGAGACAACACCGAGACGGATCCCTTGTCTGTCTGACAGTAGGGTTTTTTTTATGAAAATAGGTTTACAGAACCTGTTTGAACAAATGGCGGTTGTCTGGTTCCTAGATAATCTTCCCCAAGTAGTTTAAGTGGCTCTAAATTATCAATCTAGTTTAATAGAGGCATCTGAGATCTTGATAGATAGATATCCAGATGGTGCTTGGGAGATAATGAAGCCTTATATATGCCTGAAAGAGGTTCAAAGAGAAGCTCGGTTGAAAGTTGGTCTGGCATCTACATCATCAGAAATCTTTCTCACTGGAAAAGGACTCGGATATGCTATCAGTCAGGCTATAGCAGGAGAACTCAAGAAGATGATAGGAGATGATAGCACCCAAATTAAAAGGAGACCTTTTCGAATTCCTTTTATATCTACTACTCCAGGGATCACACTAGATAGGTGTATCTTTATATGGGTGAACTCTTGCGTACTCCTTGGTAAGTGGGATAATATGAGACAGCTAGAACTTCTTATTCAGCTAGGACGAGAATGCGTAATGCAATGCAAGACTTTAGAAGGACTTGACACGAGCAAGCTTATGATTATAATGGCAAATCTCACCAGAGGAGAACTCAGCTTTAAACATGCTATAACTAGCAGTAAGATCTATATATCCAAAGTGGGAGTAGAGCCCTGGGTTATTCCTCAGATCTTACCTGATAAGAAGAATTTCTCAGTTAGCATGACGAAGTACAACCCAAGAGTGGATGATAGAATTTTTAGAGATGCAATAATGATTATCTTAGAGCAGATAGGTCCCGCTGTCCAAGTCAACCTAGGGATAACAGACAATACCACAGAGAATACCCCCTCCCTAAGCGAATCGAGTATCCGGAGAGGAAAGCACATACAAGAGCTGATCATTATTATAGATTGGTAGGCACCTATTCCACTGCGGGGTTAAAGTACATCCAGCTATATAGTGTAAAACAGATAAGAGAAACTCTAGCGTCAGCCCACCTCGCGGAGGGAGCTGGAGAAGTAGCAAGGGCCACATCACTAGTGTTAGGTTCTCAGATCATAATCTACAACTCTCTAATTCAAGTTGGAGCTGGCTTAAATCATAGGGTGTATGATTATGTTCCAGCAGAGTTACAAGACTTAGAAGCAACAAAGGTACTAGGACCCACATTGTGTTACCAGACGGGAAGGGATATTACGAAGCAGGTTACCATTCTTAGATATAAAAATTTGATATCTGAAAAAGTTACAAGATCAGGGTGATAACGTGTGATGCTGAATTTCCCTCTGAGGGTACAATAGATCAAGCACGTGCATTAATTCAGAGTTTCCTTAGGATCAGCAGTTCATCTAAGGATGGAACTCTCTTAATATTCAAGACCTTCTGTAGATTGAGAGAAGTGCTTCTGCAGCAAGTAGGGATTTGGTCCCGATCTGTCCATAATCCTGAGATCATAGTCCCTAAGTTCTCATCTCATGAATCTTCTGAGATCTTCTTGATTGGACAGAATAAATCACTCTCTGAGCAAAGCCTCCAGTCTATGGTCATAAGCAATTATAATACTGAGATTATAGATTCATTAATTGAGATTAGGAATCCATCATTCCCATTTCTAACGCAGGAGGATAGAGATAAGTTGGACGATTTATACTCGTTCTGTGATCATCTCGGTGTGCAATACAACAACCTTGAAGCTATACACATTTTTCTGAACTTCAGCGTTCCGGAACGTGATCTAGAAGAGAACCTATTGAGTGCTATATCCCATAGCATCGATCACAACAGGATGAGGATAGATGAGAGGGCAGCCATAATCAAAAGATTTGGTGAAGCTACTGATCTGAGTCGGATGCAGAGATTGATTGTACATTCTGTGAGGCAGGAATCTAGAGAACTTCATCTCTGCTCCGAGAGTATATTCAACGCAAATATCTTGAGAGCTCTAATTGTAAAGCATGAGATCCCTGAGAGTTTCTTATTATCGGATTGGGTCATAGAGAAACAGAGTGAATGTCTTTATGTCTATACACCAAATTGGCCTGACTGGATTCAAGCATACGGGAGATCTTTCTTTAAAATT

>TRINITY_DN102665_c2_g5_i1 YP_009336924.1 RNA-dependent RNA polymerase [Hubei odonate virus 10] from the St_CH library

AATCGAATAGCAAAGAGGAACATAAAGGAAGCTATTAGTGAACTCTTCCAAAGCCTTCCGGAATTGTCTGATCAGAAGAGAGACCTGCTAAGAAGGTACGACTCAATAGAAATAACACAAACAGTTAGAGAATGGTGTGTCTACCGAAATGTTTGGGAGGCAATGAGGGATATTATGAAAGACATGCAAATTACAGATGATTACCGATTTAAAGAAGTAGCTAAAGGGTTGACCACTGGATCCTTCTTTGTCAACCCGTACTTTATGATATACCAGGAAAATCAGTATGTGTGTTGGCTACTAGATTATAATCAGGTACTGATGATCTCTGACACAGTTGGATCTAGGTTTTTATCCTTGTTTTTCTGTTCGATCCACGAAAAGATTGCAATATCACCGATGCCAAAAGTCTCTGAGATGTTGGAGTTCTATGTGACGGGGGATAACATATTGACTAAGTTTGGTAATTCTGGATACAACCATCTAAAACTCTTGGAATCTCTATCTATTGCAAGCTATCTATCCAGGTTTGAACCTCTAAAAATTAGTAAAAACTACTATCACCAACTACTTGAAGAAGCTGGAGATGATAAGGACCTATTAGAGAGTATGAAAATTTTATATGAAATCTTAACTCGAATAGGAGATCATCCCCTAAAGTTGTTTGAAGTTTATGGGTGTTTTAGACACTTTGGCCATCCTACTGTCGATGAAATAGAAGGAATCAAAAGTTTGAAAGAAAATAGTAGAATGGAGATAAAGATGGATCAAGAACAGCTACGAAAGGTTGGGGGGGGGCTTTTAATCGAATGATTATTCTAGAGTTTATCTCCACCAGAAAGAGGTGGCCAAGTGTCACCTCTCGGAACAGTGCCAGAATAGACAAATGAAAAATCTAGTTAAGAATCAGCCTGTGGCTATATCAGAGTATGATTTGGTCATCTCTCTTAATGACTGGTCATACATTGAATTTGATCAGGAGTTCCAATTCGATGATTTCCCAGACTTCACGTCTCTACTCTCAGATACTGCTATATCCCCATATCTGAGCCACTGGTACTCTGTATTTAGCAAAGACTTGTTGAATGTTTCGCATCCGACGGATATGGAAGAATCCCGTAGAGTTCTGATAGAAGTCCTCAAACGTGAAAAAATCAATTGTAAAGAGATCAGAGAAATAATTCAATGGCACAGAGTTTGCCAGTCAAAACCTAATAAATAATGAATTGCAGGCACAGAAGGTGCTTAACTGTTTTAATTATAAGTTGGAATAAACCCTGTAATTAATCCTTGCCTCTGGCCTAAGTCCAGATGGCACTCAAACCTTTAACCTATCTTAAACAAAAGAAATATTCAATAATATTTACCCATTAAGCTTGAGCGGCAAATTAAATTGTCAGAGATGACCCGGAAAACACTATGGTTTCTTTTGTCGAAGAGTTAATTCGGTCTCGTTGTGGCACTAGCAAAGAATTCTTACTGCTCACATCCGGGGTGAACTATGGCGGATCTGTACAGCACCGTTTCTCAGATGTTACATCTAAACATGAGAGTCGCCCAGGAATACGAGACAAAAATCACTCTAGGGTCATGATCTCAAGTGATCAGTTAGGAAAGTATGCTAGAGGAAAGGACAATTACTCAATAGTGTTTCAATTTTTATATCTCCAAGTGCTTCATCTTATTAATACCTTGCTAGTTTATGACCGACACATCTTGAAAGATGGAAATTTAGTTTTACACCAACACTTAGTTTGTAAGGGGTGTCTAGACCTACTTGATGAACCTCCTATCTTAAATAAATCTTGTGCTCCTGAAGTTCCCAAGCTAATGAATTGTCCACTCATATTTGCCAGTATACGACAAAAATCAGAGGCCCTACCTCAACATTGCATAGAGAATACTCCTACTGTGGAGCCTAACCCAAGAGACCCAGATATTAGGAGAAAGAGTGCTTACGCAGCTGCTCTTATAATCATA

>TRINITY_DN77321_c0_g3_i1 YP_009336924.1 RNA-dependent RNA polymerase [Hubei odonate virus 10] from the St_IT1 library

TAATCGAATAGCAAAGAGGATCATAAAGGAAGCTATTAGCGAACTCTTCCAAAGCCTTCCGGAATTGTCTGATCAGAAGAGAGACCTGCTAAGAAGGTACGACTCAATAGAAATAACACAAACAGTTAGAGAATGGTGTGTCTACCGAAATGTTTGGGAGGCAATGAGGGATATTATGAAAGACATGCAAATTACAGATGATTACCGATTTAAAGAAGTAGCTAAAGGGTTGACCACTGGATTCTTCTTTGTCAACCCGTACTTTATGATATACCAGGAAAATCAGTATGTGTGTTGGCTACTAGATTATAATCAGGTACTGATGATCTCTGACACAGTTGGATCTAGGTTTTTATCCTTGTTTTTCTGTTCGATCCACGAAAAGATTGCAATATCACCGATGCCAAAAGTCTCTGAG

**Reovirus**

>Contig7801 YP_005255241.1 S2 gene product [Spissistilus festinus reovirus] from the St_IT1 library

GGCCTTACCTATTTTATCAGTGAACACATTCTTAAATTCTGAAATAATATTGGGGTATCAAGTGATGGCAAGTATAAGACATTCACGTTAACTATTCCTTCGATCTTGCACGACCCTCCTACAATTACAGGGATCACATTTTTGTACACGAAAAATGCTTCTGGGTTAGAATTTCACCCTAAAGTTAACTATTTACCCACTTTTGATGCTATAGAATTTATGAACCGGCCTTTTTCAGAGATTGATTTTACCAGCGCCCAGTCAGTAAACGATCATATCCTAAGAGAGTTACTAACCCATTCCGTATACCAGTCACGTGTAAGCGGAATATGGCGCCAGACCGCAGCCATCCTGAGTTTGTTAAGGGCGTTCCAAATGGAGGATCACGTCGTCTATCGATACTTCAGTCTATTATTAGAGCCTTACGACGCAGCTCCATTTTTCGAGCGCAACGATCAGGTGACTTGGACAGACGGGAAAGTGAAGGCGGTGTCCGTATTACCACTACTGATTCACGGAGCGGGACAGTTAGCCATCATGGCGTTGACAGACGTAGTGAAGGAAATAGACGCACTGGTTTGTATCCAGCATTGGATGGACTGCCTACACAGAGGGTTCCAAGACGCCAAACTAGAGACACAGAAGGTGCTTCGCAACTGGCTTCCCTTAGCTCTGACGAAACTGGACATCCAAAAAGTCCCGATGTGGTCTCTGACTGGTTCGCTAGTAGGGTGGGGATGGAAGGATCCGAAGGAACCAGAAGAGCTGTTAAAGACGTTGGAACAGCAGAGAGCGCCAACTCTAGAGCTGCTGAGTCAGAGCGTGAGGAATTATCTGGATGGACTGACCCAGCAGAGCAGTTTACAATCGGTGATACGAGGTTACCACGTTGGACGCGTCATAGCCAACGATGGCACGTTCTACGCCTCCATTCCGGAAGTAACAATAGACGCAGCGGTTCTTCCCAAAGTCAATACTACGATACTCCATGCACTCTGCCCCTTGTCTCACTTGACGACGGAGCAGGTTCTAGCCTACGATCAGCTTCAGTCTCCGGAGTTCTCTCCAGGGTACAACTATTTGAGACGTGCCTTTCCAACTTGGATTACTAATTCCCGTCGTATTTTCGAGCACTTAAACCATGAGATTCCTAAGTTTTTAACAACATCTTCGGCTGGTCATATTCCAGATGATCTTCTTCCTGACTTTAGTAGCAATCGGAGGTTACAGAGCGCACGCAAGAAGAGGATAGTAGCATTTGTTGGTGAAAGTGGTGTGATGCTTAACAAAGAGGCTTTTCGAAAACGGCTTTCCGAGCCTGTCTATGCTGTTGAGCGGACTCAGATAGGTCGGAGAGCACGCGCTGTGGCTGGTGTCAACAACCCCCGGATTTTAGCATCTCTCCCATTTTTAAAGGGGGCTGAGGTATTGCAGAAGATTTTGCGTCACGCTGCGTCAGGTAAGCAGACTGGAAGTGTTTTGGATCTCGTCCGGACTTTGCATTGGAGTGTATTAAAAAACGTTCTCTTAAGTAGTATGGATGTTAATGGATTTGACGCAAGCGTTCAAATGGCTTCCCAAACACAGTTTGTGTCCATGATAGGTCAGATCTTAGGCCCCGTTACGGCAGGTAAATACTTATGCTATGAAAAAGAGACTGATCCAACGTCTTCTATCCCTAATCAAATGGTATCGCCTTTGGAACAGCTGGCTGCTGATGTGATGACTTGTTTAAATTAATGCTGACTGGTATAATTTTGAGTGAAATATAAAATGTGT

>TRINITY_DN98591_c0_g4 YP_005255241.1 S2 gene product [Spissistilus festinus reovirus] from the St_IT2 library

CTTTATTTTAAGCTCCACTTCAAAGTTAATAGCCTTACCTATTTTATCAGTGAACACATTCTTAAATTCTGAAATAATATTGGGGTATCAAGTGATGGCAAGTATAAGACATTCACGTTAACTATTCCTTCGATCTTGCACGACCCTCCTACAATTACAGGGATCACATTTTTGTACACGAAAAATGCTTCTGGGTTAGAATTTCACCCTAAAGTTAACTATTTACCCACTTTTGATGCTATAGAATTTATGAACCGGCCTTTTTCAGAGATTGATTTTACCAGCGCCCAGTCAGTAAACGATCATATCCTAAGAGAGTTACTAACCCATTCCGTATACCAGTCACGTGTAAGCGGAATATGGCGCCAGACCGCAGCCATCCTGAGTTTGTTAAGGGCGTTCCAAATGGAGGATCACGTCGTCTATCGATACTTCAGTCTATTATTAGAGCCTTACGACGCAGCTCCATTTTTCGAGCGCAACGATCAGGTGACTTGGACAGACGGGAAAGTGAAGGCGGTGTCCGTATTACCACTACTGATTCACGGAGCGGGACAGTTAGCCATCATGGCGTTGACAGACGTAGTGAAGGAAATAGACGCACTGGTTTGTATCCAGCATTGGATGGACTGCCTACACAGAGGGTTCCAAGACGCCAAACTAGAGACACAGAAGGTGCTTCGCAACTGGCTTCCCTTAGCTCTGACGAAACTGGACATCCAAAAAGTCCCGATGTGGTCTCTGACTGGTTCGCTAGTAGGGTGGGGATGGAAGGATCCGAAGGAACCAGAAGAGCTGTTAAAGACGTTGGAACAGCAGAGAGCGCCAACTCTAGAGCTGCTGAGTCAGAGCGTGAGGAATTATCTGGATGGACTGACCCAGCAGAGCAGTTTACAATCGGTGATACGAGGTTACCACGTTGGACGCGTCATAGCCAACGATGGCACGTTCTACGCCTCCATTCCGGAAGTAACAATAGACGCAGCGGTTCTTCCCAAAGTCAATACTACGATACTCCATGCACTCTGCCCCTTGTCTCACTTGACGACGGAGCAGGTTCTAGCCTACGATCAGCTTCAGTCTCCGGAGTTCTCTCCAGGGTACAACTATTTGAGACGTGCCTTTCCAACTTGGATTACTAATTCCCGTCGTATTTTCGAGCACTTAAACCATGAGATTCCTAAGTTTTTAACAACATCTTCGGCTGGTCATATTCCAGATGATCTTCTTCCTGACTTTAGTAGCAATCGGAGGTTACAGAGCGCACGCAAGAAGAGGATAGTAGCATTTGTTGGTGAAAGTGGTGTGATGCTTAACAAAGAGGCTTTTCGAAAACGGCTTTCCGAGCCTGTCTATGCTGTTGAGCGGACTCAGATAGGTCGGAGAGCACGCGCTGTGGCTGGTGTCAACAACCCCCGGATTTTAGCATCTCTCCCATTTTTAAAGGGGGCTGAGGTATTGCAGAAGATTTTGCGTCACGCTGCGTCAGGTAAGCAGACTGGAAGTGTTTTGGATCTCGTCCGGACTTTGCATTGGAGTGTATTAAAAAACGTTCTCTTAAGTAGTATGGATGTTAATGGATTTGACGCAAGCGTTCAAATGGCTTCCCAAACACAGTTTGTGTCCATGATAGGTCAGATCTTAGGCCCCGTTACGGCAGGTAAATACTTATGCTATGAAAAAGAGACTGATCCAACGTCTTCTATCCCTAATCAAATGGTATCGCCTTTGGAACAGCTGGCTGCTGATGTGATGACTTGTTTAAATTAATGCTGACTGGTATAATTTTGAGTGAAATATAAAATGTGTGATTACTGCATATTGAAGGTGAAATAAAATGTGAACTTTGTTGTATAGTATTAAATGAAAGTGTA

>TRINITY_DN104045_c1_g9 YP_005255241.1 S2 gene product [Spissistilus festinus reovirus] from the St_CH library

GTTAAGTCCTTTATTTTAAGCTCCACTTCAAAGTTAATAGCCTTACCTATTTTATCAGTGAACACATTCTTAAATTCTGAAATAATATTGGGGTATCAAGTGATGGCAAGTATAAGACATTCACGTTAACTATTCCTTCGATCTTGCACGACCCTCCTACAATTACAGGGATCACATTTTTGTACACGAAAAATGCTTCTGGGTTAGAATTTCACCCTAAAGTTAACTATTTACCCACTTTTGATGCTATAGAATTTATGAACCGGCCTTTTTCAGAGATTGATTTTACCAGCGCCCAGTCAGTAAACGATCATATCCTAAGAGAGTTACTAACCCATTCCGTATACCAGTCACGTGTAAGCGGAATATGGCGCCAGACCGCAGCCATCCTGAGTTTGTTAAGGGCGTTCCAAATGGAGGATCACGTCGTCTATCGATACTTCAGTCTATTATTAGAGCCTTACGACGCAGCTCCATTTTTCGAGCGCAACGATCAGGTGACTTGGACAGACGGGAAAGTGAAGGCGGTGTCCGTATTACCACTACTGATTCACGGAGCGGGACAGTTAGCCATCATGGCGTTGACAGACGTAGTGAAGGAAATAGACGCACTGGTTTGTATCCAGCATTGGATGGACTGCCTACACAGAGGGTTCCAAGACGCCAAACTAGAGACACAGAAGGTGCTTCGCAACTGGCTTCCCTTAGCTCTGACGAAACTGGACATCCAAAAAGTCCCGATGTGGTCTCTGACTGGTTCGCTAGTAGGGTGGGGATGGAAGGATCCGAAGGAACCAGAAGAGCTGTTAAAGACGTTGGAACAGCAGAGAGCGCCAACTCTAGAGCTGCTGAGTCAGAGCGTGAGGAATTATCTGGATGGACTGACCCAGCAGAGCAGTTTACAATCGGTGATACGAGGTTACCACGTTGGACGCGTCATAGCCAACGATGGCACGTTCTACGCCTCCATTCCGGAAGTAACAATAGACGCAGCGGTTCTTCCCAAAGTCAATACTACGATACTCCATGCACTCTGCCCCTTGTCTCACTTGACGACGGAGCAGGTTCTAGCCTACGATCAGCTTCAGTCTCCGGAGTTCTCTCCAGGGTACAACTATTTGAGACGTGCCTTTCCAACTTGGATTACTAATTCCCGTCGTATTTTCGAGCACTTAAACCATGAGATTCCTAAGTTTTTAACAACATCTTCGGCTGGTCATATTCCAGATGATCTTCTTCCTGACTTTAGTAGCAATCGGAGGTTACAGAGCGCACGCAAGAAGAGGATAGTAGCATTTGTTGGTGAAAGTGGTGTGATGCTTAACAAAGAGGCTTTTCGAAAACGGCTTTCCGAGCCTGTCTATGCTGTTGAGCGGACTCAGATAGGTCGGAGAGCACGCGCTGTGGCTGGTGTCAACAACCCCCGGATTTTAGCATCTCTCCCATTTTTAAAGGGGGCTGAGGTATTGCAGAAGATTTTGCGTCACGCTGCGTCAGGTAAGCAGACTGGAAGTGTTTTGGATCTCGTCCGGACTTTGCATTGGAGTGTATTAAAAAACGTTCTCTTAAGTAGTATGGATGTTAATGGATTTGACGCAAGCGTTCAAATGGCTTCCCAAACACAGTTTGTGTCCATGATAGGTCAGATCTTAGGCCCCGTTACGGCAGGTAAATACTTATGCTATGAAAAAGAGACTGATCCAACGTCTTCTATCCCTAATCAAATGGTATCGCCTTTGGAACAGCTGGCTGCTGATGTGATGACTTGTTTAAATTAATGCTGACTGGTATAATTTTGAGTGAAATATAAAATGTGTGATTACTGCATATTGAAGGTGAAATAAAATGTGAA

>TRINITY_DN92592_c0_g1 YP_005255241.1 S2 gene product [Spissistilus festinus reovirus] from the St_FR library

CAAAGTTAATAGCCTTACCTATTTTATCAGTGAACACATTCTTAAATTCTGAAATAATATTGGGGTATCAAGTGATGGCAAGTATAAGACATTCACGTTAACTATTCCTTCGATCTTGCACGACCCTCCTACAATTACAGGGATCACATTTTTGTACACGAAAAATGCTTCTGGGTTAGAATTTCACCCTAAAGTTAACTATTTACCCACTTTTGATGCTATAGAATTTATGAACCGGCCTTTTTCAGAGATTGATTTTACCAGCGCCCAGTCAGTAAACGATCATATCCTAAGAGAGTTACTAACCCATTCCGTATACCAGTCACGTGTAAGCGGAATATGGCGCCAGACCGCAGCCATCCTGAGTTTGTTAAGGGCGTTCCAAATGGAGGATCACGTCGTCTATCGATACTTCAGTCTATTATTAGAGCCTTACGACGCAGCTCCATTTTTCGAGCGCAACGATCAGGTGACTTGGACAGACGGGAAAGTGAAGGCGGTGTCCGTATTACCACTACTGATTCACGGAGCGGGACAGTTAGCCATCATGGCGTTGACAGACGTAGTGAAGGAAATAGACGCACTGGTTTGTATCCAGCATTGGATGGACTGCCTACACAGAGGGTTCCAAGACGCCAAACTAGAGACACAGAAGGTGCTTCGCAACTGGCTTCCCTTAGCTCTGACGAAACTGGACATCCAAAAAGTCCCGATGTGGTCTCTGACTGGTTCGCTAGTAGGGTGGGGATGGAAGGATCCGAAGGAACCAGAAGAGCTGTTAAAGACGTTGGAACAGCAGAGAGCGCCAACTCTAGAGCTGCTGAGTCAGAGCGTGAGGAATTATCTGGATGGACTGACCCAGCAGAGCAGTTTACAATCGGTGATACGAGGTTACCACGTTGGACGCGTCATAGCCAACGATGGCACGTTCTACGCCTCCATTCCGGAAGTAACAATAGACGCAGCGGTTCTTCCCAAAGTCAATACTACGATACTCCATGCACTCTGCCCCTTGTCTCACTTGACGACGGAGCAGGTTCTAGCCTACGATCAGCTTCAGTCTCCGGAGTTCTCTCCAGGGTACAACTATTTGAGACGTGCCTTTCCAACTTGGATTACTAATTCCCGTCGTATTTTCGAGCACTTAAACCATGAGATTCCTAAGTTTTTAACAACATCTTCGGCTGGTCATATTCCAGATGATCTTCTTCCTGACTTTAGTAGCAATCGGAGGTTACAGAGCGCACGCAAGAAGAGGATAGTAGCATTTGTTGGTGAAAGTGGTGTGATGCTTAACAAAGAGGCTTTTCGAAAACGGCTTTCCGAGCCTGTCTATGCTGTTGAGCGGACTCAGATAGGTCGGAGAGCACGCGCTGTGGCTGGTGTCAACAACCCCCGGATTTTAGCATCTCTCCCATTTTTAAAGGGGGCTGAGGTATTGCAGAAGATTTTGCGTCACGCTGCGTCAGGTAAGCAGACTGGAAGTGTTTTGGATCTCGTCCGGACTTTGCATTGGAGTGTATTAAAAAACGTTCTCTTAAGTAGTATGGATGTTAATGGATTTGACGCAAGCGTTCAAATGGCTTCCCAAACACAGTTTGTGTCCATGATAGGTCAGATCTTAGGCCCCGTTACGGCAGGTAAATACTTATGCTATGAAAAAGAGACTGATCCAACGTCTTCTATCCCTAATCAAATGGTATCGCCTTTGGA

>TRINITY_DN123868_c0_g5 YP_005255241.1 S2 gene product [Spissistilus festinus reovirus] from the St_HU library

GTTAAGTCCTTTATTTTAAGCTCCACTTCAAAGTTAATAGCCTTACCTATTTTATCAGTGAACACATTCTTAAATTCTGAAATAATATTGGGGTATCAAGTGATGGCAAGTATAAGACATTCACGTTAACTATTCCTTCGATCTTGCACGACCCTCCTACAATTACAGGGATCACATTTTTGTACACGAAAAATGCTTCTGGGTTAGAATTTCACCCTAAAGTTAACTATTTACCCACTTTTGATGCTATAGAATTTATGAACCGGCCTTTTTCAGAGATTGATTTTACCAGCGCCCAGTCAGTAAACGATCATATCCTAAGAGAGTTACTAACCCATTCCGTATACCAGTCACGTGTAAGCGGAATATGGCGCCAGACCGCAGCCATCCTGAGTTTGTTAAGGGCGTTCCAAATGGAGGATCACGTCGTCTATCGATACTTCAGTCTATTATTAGAGCCTTACGACGCAGCTCCATTTTTCGAGCGCAACGATCAGGTGACTTGGACAGACGGGAAAGTGAAGGCGGTGTCCGTATTACCACTACTGATTCACGGAGCGGGACAGTTAGCCATCATGGCGTTGACAGACGTAGTGAAGGAAATAGACGCACTGGTTTGTATCCAGCATTGGATGGACTGCCTACACAGAGGGTTCCAAGACGCCAAACTAGAGACACAGAAGGTGCTTCGCAACTGGCTTCCCTTAGCTCTGACGAAACTGGACATCCAAAAAGTCCCGATGTGGTCTCTGACTGGTTCGCTAGTAGGGTGGGGATGGAAGGATCCGAAGGAACCAGAAGAGCTGTTAAAGACGTTGGAACAGCAGAGAGCGCCAACTCTAGAGCTGCTGAGTCAGAGCGTGAGGAATTATCTGGATGGACTGACCCAGCAGAGCAGTTTACAATCGGTGATACGAGGTTACCACGTTGGACGCGTCATAGCCAACGATGGCACGTTCTACGCCTCCATTCCGGAAGTAACAATAGACGCAGCGGTTCTTCCCAAAGTCAATACTACGATACTCCATGCACTCTGCCCCTTGTCTCACTTGACGACGGAGCAGGTTCTAGCCTACGATCAGCTTCAGTCTCCGGAGTTCTCTCCAGGGTACAACTATTTGAGACGTGCCTTTCCAACTTGGATTACTAATTCCCGTCGTATTTTCGAGCACTTAAACCATGAGATTCCTAAGTTTTTAACAACATCTTCGGCTGGTCATATTCCAGATGATCTTCTTCCTGACTTTAGTAGCAATCGGAGGTTACAGAGCGCACGCAAGAAGAGGATAGTAGCATTTGTTGGTGAAAGTGGTGTGATGCTTAACAAAGAGGCTTTTCGAAAACGGCTTTCCGAGCCTGTCTATGCTGTTGAGCGGACTCAGATAGGTCGGAGAGCACGCGCTGTGGCTGGTGTCAACAACCCCCGGATTTTAGCATCTCTCCCATTTTTAAAGGGGGCTGAGGTATTGCAGAAGATTTTGCGTCACGCTGCGTCAGGTAAGCAGACTGGAAGTGTTTTGGATCTCGTCCGGACTTTGCATTGGAGTGTATTAAAAAACGTTCTCTTAAGTAGTATGGATGTTAATGGATTTGACGCAAGCGTTCAAATGGCTTCCCAAACACAGTTTGTGTCCATGATAGGTCAGATCTTAGGCCCCGTTACGGCAGGTAAATACTTATGCTATGAAAAAGAGACTGATCCAACGTCTTCTATCCCTAATCAAATGGTATCGCCTTTGGAACAGCTGGCTGCTGATGTGATGACTTGTTTAAATTAATGCTGACTGGTATAATTTTGAGTGAAATATAAAATGTGTGATTACTGCATATTGAAGGTGAAATAAAATGTGAA

**Phlebovirus**

>Contig1152 AFN73039.1 glycoprotein, partial [FinV707 virus] from the St_FR library

GTTAAAAGGTTCTTTTGAGTTCGGACCCCGTTTAGGCGGTGTTGTCGAGTCTGAGTGCCTTTAGAAAGGTTAAGTTATTGTGAAATTTGTTTTCTTATTGTTGTTGTTGTTTCGAGGCGTTAAATTATGGCTGTGGAGAAGAGAGCCGCTAACCGTCGTATCAGTGGACAGGTCCCCACCGCATTTTGAAGTTTCTCACTCCAGTGACGGCCCGGTTGGGAAATTCCCAGACCGGGAAGGTTTTCCGGAGGGTGCACATTTCACACCTTAAACCTTACCGTGGATATATTTAAATGCTCGATTGTTTCTTGGCGGGGAGTGCGCTGGACCAGGGAGTCTGATAAATTCTCTGTCCCGTTCACTTGTTTGGTTCCCTTTTCTTTTCACTATTTTGCTGGCCAGCACAATTTCCCTGGGGTTGAATAGTCCCTCAGTATTTGGGGATTCTTTGACGTCCCCGCCTTGAACTTTCGAGCAGCCATTGGGTGTGTTTTCTCCTAGAATGTTTTCTCCAAAAATCGGTGAGTATAACAAACTAATGTACAGAGGAATCTGCCAAAGCTGCCTTACACATTTGTATGGAATTGAATTATTTGACAAGACTCATGTGCAAATTACTCAAGGATTCACTTAAGACCCAAGCTATATGTTCGATGATAGCTGTCAAGGAGAACTTCAGATGCCGTCTGGATGCTCCAGACTGAACAATATTGGGACAGTTAATCAGATCTCATGCTCGAATGAATCGTTGAGATCATCTGATATTGAAGCATTCTCGGATATGAATCATGAAATCACTGATTTTGTGTGCTCTATAGGAAAGGTGCAATTTAGAGAATGTCAAACAATGACTGAGATGTGGTCAACAATTGACATACTGATAGTGAAGGATCAATATAAAATAATTGATAATCCTCAATTGGCATACTTTCGTAATGTGAGGAGTCCAGAATCATACCATTCAAATATAGATGGCTGTCACAATTGCTTTGTAAAGAGAGGATCATGTAGAGGAGATACAGTCTTATGCCAAAACTTTGGCTGCACCTCGTCGAAAGGCACCTCTTGTCACATTACTTATGGTGATGACAAGAATTACCTGTCTTATACTAATTCACAAGGAAAATCATTGTTTGTGCACTGCCATGCTACACTAGATGTTAGAATGAAAGTGGTACAACGACGGAAAGCTGAAGAGAAGGAGTCAGTGAGTCTTAAATCTAATCTTGTTTATGATTGCACTGACACTGGATTTTCTATAACAAGGAAGTCAATTGATGTGCACATCTTGAAAGTAATGTACTGCATTGAGGATTTTTGTGCCACAATGAGAATCTTTGATTCCACCAAAATCATAAATAATGTTACTGTACTATCCCAAATCACTTCCTCACTTCTCAGCACACATTAATTGCA

>TRINITY_DN90923_c0_g1 AFN73039.1 glycoprotein, partial [FinV707 virus] from the St_IT1 library

GTCCCGCCCACTTGTTTGCTTCCCCCTTCCCTTCAACACCTTGCTGGCCAGCACAATTTCCCTGGGGTTGAATAGTCCCTCAGTATTTGGGGATTCTTTGACGTCCCCGCCTTGAACTTTCGAGCAGCTATTGGGTGTGTTTTCTCCTAGAATGTTTTCTCCAAAAATCGGTGAGTATAACAAACTAATGTACAGAGGAATCTGCCAAAGCTGCCTTACACATTTGTATGGAATTGAATTATTTGACAAGACTCATGTGCAAATTACTCAAGGATTCACTTAAGACCCAAGCTATATGTTCGATGATAGCTGTCAAGGAGAACTTCAGATGCCGTCTGGATGCTCCAGACTGAACAATATTGGGACAGTTAATCAGATGTCATGCTCGAATGAATCGTTGAGATCATCTGATATTGAAGCATTCTCGGATATGAATCATGAAATCACTGATTTTGTGTGCTCTATAGGAAAGGTGCAATTTAGAGAATGTCAAACAATGACTGAGATGTGGTCAACAATTGACATACTGATAGTGAAGGATCAATATAAAATAATTGATAATCCTCAATTGGCATACTTTCGTAATGTGAGGAGTCCAGAATCATACCATTCAAATATATATGACAGCCATATAAATATATGGCTGTCACAATTGCTTTGTA

>Contig15563 from the St_CH library

CAAGGGTTCACACAAGACCCAAGCTATATGTTCGATGATAGCTGTCAAGGAGAACTTCAGATGCCGTCTGGATGCTCCAGACTGAACAATATTGGGACAGTTAATCAGATCTCATGCTCGAATGAATCGGTGAGATCATCTGATATTGAAGCATTCTCGGATATGAATCATGAAATCACTGATTTTGTGTGCTCTATAGGAAAGGTGCAATTTAGAGAATGTCAAACAATGACTGAGATGTGGTCAACAATTGACATACTGATAGTGAAGGATCAATATAAAATAATTGATAATCCTCAATTGGCATACTTTCGTAATGTGAGGAGTCCAGAATCATACCATTCAAATATATATGACAGCCATATAAATATATGGCTGTCACAATTGCTTTGTAAAGAGAGGATCATGTAGAGGAGATACAGTCTTCTGCCAAAACTTTGGCTGCACCTCGTCGAAAGGCACCTCTTGTCACATTACTTATGGTGATGACAAGAATTACCTGTCTTATACTAATTCACAAGGAAAATCATTGTTTGTGCACTGCCATGCTACACTAGATGTTAGTATGAAAGTGGTACAACGACGGAAAGCTGAAGAGAAGGAGTCAGTGAGTCTTAAATCTAATCTTGTTTATGATTGTACTGACACTGGATTTTCTATAACAAGGAAGTCAATTGATGTGCACATCTTGAAAGTAATGTACTGCATTGAGGATTTTTGTGCCACAATGAGAATCTTTGATTCCACCAAAATCATAAATAATGTTACTGTACTATCCCAAATCACTTCCTCACTTCTCAGCACACAATAATTGCAAAAACCTTTATTCAAAATCAGTATAAGCCCCTGAAGGAAGTTATTGTATGCTTGGCCAAAGATTTTTGCAAGACAATTAAATGCCATCTATGTCTAGAGAGGTATTTGAACACAAACTGTTAGACCTACCTGGAACATATTGCACTTGTCTCAGGAGTGATAATAGGATCAGTTTTGTTAGTTGCTGTAGCAATAATATTCCATGCATTAGGTACCATACTTCAAATGTGCTATCTGGTTGTGAAGAAAGTATATGGATTCACCAAGGTCTCGATTATGTCTATTTGCTGGGCATTCAGTTTATTTCTAATGAGGGGTAGTAAGGCACAGAATAAGCCCCGTAAAAGTAAGAATGACACAACTCCTCTACTTGTATCTGAGAGTGACGAGGAAGACAAATCAGAACCTGAAGCAAAGATAAGGCAGTCTGAAAGAGTTAAGCAACGGATGAAGAACAATCAGGCAGGAACTGGCAAACCAACCACAGATGCAAGAGGGAAAAGGTTGCTTAGCTTGAAAGAGTTGATGGCTCTACCAGCTATCATTTGCTTTGGCTTCATAATACCTGGACTAATACAACCAGGACAAGCTTGCTCCCAAGTCACTAGTCTTATTGCAACTACTAAAAACTGTGAATTCTATGCCAGTGGCGCCCGTAGATCCTGTTCAATTAATGTGCAGGCCATCATGAGATTGAATGGTATTGGAGTTGAATCTTGTTTGGCCTTGTCTGGAATAAATATGGGCATGCTAAAAATTAGCATGAGGAAGGTAAAAGATGTATGTTATCCGGGAGAAACCTATTTTGTGCCAATGGTGAGCTAGAAGGTAAGATCTTATTCTGTGTGCCACCTAACAGAGTTCTGTGGAGTTGACAAATATGAGGAAGCAAACAATGATGGCGATGTGCCCTTCAAATATGAAGACGGATTGGGTCAAAACTATTGTAGCAGATCCTCTAGTTGGTTCACAGGTGAGTTCTGTGCGTCACCTGCTAATCTCTGTCTATACTATAGAATAATGTTGGAGAATAAGAGTGGCAGGTGGTGTCAAGTGTATAGCTGCACCAAGTGGACAACTAAGGTTGAGATAAAAGTGGAATTGCTACAAGAAGAGATATCTAAAACTGAAAGCAAAGAATTATCTCTCATTCATGGATTGACAACAAGTTTCGGCGACAAAATTAAACCATCTATTCAAGGTCTGGACCGATTCTACCATATTGATCTTCAGAAGAAGTTTGTCAAGAATGAAGATGGGATCTTTTACACCAGTGCCAATGAGAAAGGGATATTCCTAAAAGGTGCCATAGGTGAGGTTCAATGCAGTTAAGAGGAAGAGGCAAAGATAGCATCCTCTACATGTTTTTTTGACAAACACATTCTGAAGACAACTATCCTAGATGGCAGAATCATAGATGAGACGACCATTGTTGATGTTGATAAGGCATAGAGGAACCAGAATAAGTTGCCCCTGTATCTAAATGCTATTTTAGTTGACATAGAGAATGACGTTCCTGTTGTGGATATAGCGAAAGACACCCAGTTATCAATTAATGTTGAGCTTAAGAATCTAGTTGTATCTATGGCAAGCATGTCAGTAAAGTGCAACTTTGATTTTAAAGAACTAAAAGGTTGTGTAGGCTGTGCTTCAGGAGCAGACGTAAAACTAATAGGCAGTGCAAACATTGACCAAGCCACTGACACCAGGGAGACCCGGCCAGTCCAGTACACCAGGGGCATGCGCAACCACGCTCTGCTACCAATTGTAGCGCCGGGAAAAAGGATTTAGACTTATGGGGTTATAAAATTGAGATAAACTGAT

>Contig13839 from the St_IT2 library

CGGTCATTGTTAGCTTGGGTCACCTGGAAACCGGCGTTGTGTTTTGCAAGGCTCATCCAGCCAGTTCGGCTGTGGACAAGAGAGCCGCTAAGCTGTCGTATCGGTGGACAGGTCCCCACCACATTTTGAAGTTTCTCACTCCAGTGACGGCCCGGTTGGGAGATTCGCAGACCGGGAAGGTTTTCCGGAGGGTGCACATTTCTCACCTTAAACCTTACCGTGGATATATTTAAATGCTCGATTGTTTCTTGGCGGGGAGTGCGCTGGACCAGGGAGTCTGATCAACTTTCTGTCCCGTTCACTTGTTTGTAAATTCTCTGTCCCGTTCACTTGTTTGGTTCCCTTTTCTTTTCACTATTTTGCTGGCCAGCACAATTTCCCTGGGGTTGAATAGTCCCTCAGTATTTGGGGATTCTTTGACGTCCCCGCCTTGAACTTTCGAGCAGCCATTGGGTGTGTGTTTTTTCTCCTAGAATGTTTTCTCCAAAAATCGGTGAGTATAACAAAACTAATGTACAGAGGAATCTGCCAAAGCTGCCTTACACATTTGTATGGAATTGAATTATTTGACAAGACTCATGTGCAAATTACTCAAGGATTCACTTAAGACCCAAGCTATATGTTCGATGATAGCTGTCAAGGAGAACTTCAGATGCCGTCTGGATGCTCCAGACTGAACAATATTGGGACAGTTAATCAGATCTCATGCTCGAATGAATCGGTGAGATCATCTGATATTGAAGCATTCTCGGATATGAATCATGAAATCCCTGATTTTGTGTGCTCTATAGGAAAGGTGCAATTTAGAGAATGTCAAACAATGACTGAGATGTGGTCAACAATTGACATACTGATAGTGAAGGATCAATATAAAATAATTGATAATCCTCAATTGGCATACTTTCGTAATGTGAGGAGTCCAGAATCATACCATTCAAATATATATGACAGCCATATAAATATATGGCTGTCACAATTGCTTTGTAAAGAGAGGATCATGTAGAGGAGATACAGTCTTCTGCCAAAACTTTGGCTGCACCTCGTCGAAAGGCACCTCTTGTCACATTACTTATGGTGATGACAAGAATTACCTGTCTTATACTAATTCACAAGGAAAATCATTGTTTGTGCACTGCCATGCTACACTAGATGTTAGTATGAAAGTGGTACAACGACGGAAAGCTGAAGAGAAGGAGTCAGTGAGTCTTAAATCTAATCTTGTTTATGATTGTACTGACACTGGATTTTCTATAACAAGGAAGTCAATTGATGTGCACATCTTGAAAGTAATGTACTGCATTGAGGATTTTTGTGCCACAATGAGAATCTTTGATTCCACCAAAATCATAAATAATGTTACTGTACTATCCCAAATCACTTCCTCACTTCTCAGCACACAATAATTGCAAAAACCTTTATTCAAAATCAGTATAAGCCCCTGAAGGAAGTTATTGTATGCTTGGCCAAAGATTTTTGCAAGACAATTAAATGCCATCTATGTCTAGAGAGGTATTTGAACACAAACTGTTAGACCTACCTGGAACATATTGCACTTGTCTCAGGAGTGATAATAGGATCAGTTTTGTTAGTTGCTGTAGCAATAATATTCCATGCATTAGGTACCATACTTCAAATGTGCTATCTGGTTGTGAAGAAAGTATATGGATTCACCAAGGTCTCGATTATGTCTATTTGCTGGGCATTCAGTTTCTTTCTAAGGAGGGGTAGTAAGGCACAGAATAAGCCCCGTAAAAGTAAGAATGACTCAACTCCTCTACTTGTATCTGAGAGTGACGAGGAAGACAAATCAGAACCTGAAGCAAAGATAAGGCAGTCTGAAAGAGTTAAGCAACGGATGAAGAACAATCAGGCAGGAACTGGCAAACCAACCACAGATGCAAGAGGGAAAAAGTTGCTTAGCTTGAAAGAGTTGATGGCTCTACCAGCTATCATTTGCTTTGGCTTCATAATACCTGGACTAATACAACCAGGACAAGCTTGCTCCCAAGTCACTAGTCTTATTGCAACTACTAAAAACTGTGAATTCTATGCCAGTGGCGCCCGTAGATCCTGTTCAATTAATGAGCAGGCCATCATGAGATTGAATGGTATTGGAGTTGAATCTTGTTTGGCCTTGTCTGGAATAAATATGGGCATGCTAAAAATTAGCATGAGGAAGGTAAAAGATGTATGTTATCCGGGAGAAACCTATTTTGTGCCAATGGTGAGCTAGAAGGTAAGATCTTATTCTGTGTGCCACCTAACAGAGTTCTGTGGAGTTGACAAATATGAGGAAGCAAACAATGATGGCGATGTGCCCTTCAAATATGAAGACGGATTGGGTCAAAACTATTGTAGCAGATCCTCTAGTTGGTTCACAGGTGAGTTCTGTGCGTCACCTGCTAATCTCTGTCTATACTATAGAATAATGTTGAAGAATAAGAGTGGCAGGTGGTGTCAAGTGTATAGCTGCACCAAGTGGACAACTAAGGTGGAGATAAAAGTAGAATTGCTACAAGAAGAGATATCTAAAACTGAAAGCAAAGAAATATCTCTCATTCATGGATTGACAACAAGTTTCGGCGACCAAATTAAACTATCTATTCAAGGTCTTGACCGATTCTACCATATTGATCTTCAGAAGACGTTTGTCAAGAATGAAGATGGGATCTTTTACACCAGTGCCAATGAGAAAGGGATATTCCTAAAAGGTGCCATAGGTGAGGTTCAATGCAGTTAAGAGGAAGAGGCAAAGATTGTATCCTCTACATGTTTTTTTGACAAACACATTCTGAAGACAACTATCCTAGATGGAAGAATCATAGATGAGACGACCATTGTTGATGTTGATAAGGCATTGAGGGACCAGAATAAGTTGCCCCTGTATCTAAATGATATTTTAGTTGACATAGAGAATGACGTTCCTGTTGTGGATATAGCGAAATACACCCAGTTATCAATTAATGTTGAGCTTAAGAATCTAGTTGTATCTATGGCAAGCATGTCAGTAAAGTGCAACTTTGATTTTAAAGAACTAAAAGGTTGTGTAGGCTGTGCTTCAGGAGCAGACGTAAAACTAATAGGCAGTGCAAACATTGACCAAGCCACTGACACCAGGGAGACCCGGCCAGTCCAGTACACCAGGGGCATG

>TRINITY_DN121632_c2_g2 from the St_HU library

CCCGCCTTGAACTTTCGAGCAGCTATTGGGTGTGTTTTCTCCTAGAATGTTTTCTCCAAAAATCGGTGAGTATAACAAACTAATGTACAGAGGAATCTGCCAAAGCTGCCTTACACATTTGTATGGAATTGAATTATTTGACAAGACTCATGTGCAAATTACTCAAGGATTCACTTAAGACCCAAGCTATATGTTCGATGATAGCTGTCAAGGAGAACTTCAGATGCCGTCTGGATGCTCCAGACTGAACAATATTGGGACAGTTAATCAGATCTCATGCTCGAATGAATCGGTGAAATCATCTGATATTGAAGCATTCTCGGATATGAATCATGAAATCCCTGATTTTGTGTGCTCTATAGGAAAGGTGCAATTTAGAGAATGTCAAACAATGACTGAGATGTGGTCAACAATTGACATACTGATAGTGAAGGATCAATATAAAATAATTGATAATCCTCAATTGGCATACTTTCGTAATGTGAGGAGTCCAGAATCATACCATTCAAATATATATGACAGCCATATAAATAGATGGCTGTCACAATTGCTTTGTAAAGAGAGGATCATGTAGAGGAGATACAGTCTTCTGCCAAAACTTTGGCTGCACCTCGTCGAAAGGCACCTCTTGTCACATTACTTATGGTGATGACAAGAATTACCTGTCTTATACTAATTCACAAGGAAAATCATTGTTTGTGCACTGCCATGCTACACTAGATGTTAGTATGAAAGTGGTACAACGACGGAAAGCTGAAGAGAAGGAGTCAGTGAGTCTTAAATCTAATCTTGTTTATGATTGTACTGACACTGGATTTTCTATAACAAGGAAGTCAATTGATGTG

**Toti-like virus**

>TRINITY_DN95534_c0_g1_i1 YP_003800000.1 proline-alanine-rich protein [Spissistilus festinus virus 1] from the St_CH library

TATTGAGGTGCCTGTGCAACTTTGGGGCACTGTTTTCCCAACCCACTTTGGGCCCATCCAGATTGGAGATCCCCCCATTCGCCACAATCTCCATGTGCATGTGGGAGCTGCCCGCAAGCTTGGGAGGTTCACCTCCATGGCTTTCGGCTCTTTCCTCCAAGGAGGGGTCCCCAGTGAGAGGGCGGTTTCATCCGCTATTATAACTAAGTTGTCCTTTGGGAACATTAATGCAGCACGTAACCTCACCTCTTTCATCTTATCATCTTGGGATTACTACGACAACAGCTACATGTACGCCAAGCTGTGGTACTATGTGCTGCTAAATGACCTAATTGCATCTGTGGACGGGACACCCACTGCAATTGCCTTTTCTCAAAACCCAGTTGTACCCAACTGGATCAACATTGCCGACCAGAATTTGGATGTCAATGCAGTCCCAAATGCCATCTTACGGCGAGATATCATCTTAATTGATAATAAAGACATTAATGCCACCAACGCAACTGATTTACAGTTTGTTTATTGGCTCACCAACTCGGGGATGCGCTATGACGGACCACAGGGGTCTCCAACTCCACATGCCATTTATGTGGAATGGTCTAGCATCCCTATCACCGTTATATCACACGTCCAAGCCCCCAGTGCACCCCAGGCCGCCATCATCACCTCTCCCAGCTTGATTGCGATCATACATAGATTAGCTAGTAGCAGGGTAGAGTGGACATCTGCGCTGAAGGGGCTTTATTTAGCCATGGATATATTGGGGATGCGTTTGGTCAATTTTGATGACCAGTGGTGGCCCATTCGATCCAACCTGTCTGCACACAACCCCTGGATCCCTGCTGCTTGCGACTACAACTTCTTGTTACGGCTTCTCCTCATCTATCCAGATGATAATGAGGATGCTAAGTACGAAGTCTATGCCTTTACTTCGGCCACTGTGGCCACAAGGGTTAGGGTAGCCGCTCTGTATAATGCTGTGATTAGCTCTACGGCTACCACGTTGTTATACGACTTGAATATCACAGCAGGCCACTTATTGCAGCATGCCACTGGGGCTACGGACACCCCCCCAACCATCCATCAAATAATGGCAGAGGTACTGAACAACTACGATTGGTCCACCGCCCCAAATGAGCCCTTCTTCATGGCGCAGTGCCGGAGGGCCTTCCCCACTTGGATAGGGGCTAGTGTCTGCGCTAACCTGTACCCTGGGGATACCTGGGCTGGCGTGTTTAACAACAATGCCGGGGCAGCAGTGGTATACCAGCTGTTTCCGCAGGACATAACTCCTGCCTTCTACAACCCCCTGGTTCTAGTGGATTGGCTCCAAGTTCTCCCATTCGAATGGGGGGTGTGTGGTGTGAAGCCGACCCTGAATCTAAAGTCAGAAATCAGGTTGGTGGGGCCTGCCGCTCAACAGGGATGGTACAGTAGCAAGGGCAGTGCCCTGTACACCGAGCGGGCCAGTGGGAATTTCCCCATGAAGGTGGTTGCGTATGGAGCCCAAGTGATCAATGCGCTGACCCAGCAGTTTAGGATTGCCGCGGGCGCAGCCCCCACTGTTACCGCTCAGGCTGCGGACTGGATCCCAAAAGAAGGCGATGGCAAGCACAACATCATGTGGTGTGCCCCGGTCGCGGTGTCCGCAGCCCAGTTAGTGTACAACGGTGACATCCACACTTATAGGCCATGTAGTTTCATGACCTATAGTTACGCCGATGCCCAAGTTCGGGCCCCTTGCCTGGTTGGTGATGCCTTGCGACCTAATGTAGCTACGCGGTTGATAGTGTGGGTTGGCCAAGCTGCGGAGATGGCTGGGGTCTTCTTGCCCAATGTCAGAGTTACTGCCGCGCCCTCTGCCTGCCGCACCCTTTGCCTGCCCCGCTATCTCAACCTCCAACGGAGAGGAGAACTCAAGGAGCAGAAGCCCCCTGCAAGGGAGGATGCCACTGCCACCTACAGCCAAATGTGGGCACCTGAGGGGGCTCCTCTCTCAGGGGTTGGAGGAGAAGTGGCCTCTGCCCCCCGGCAACCCCTTAATACGGGCCTCCCCCCTTAGTCAGCCAGTCGCTAGGCCGGTAGCGCTGGAAGACCTTGTACATACCGTTTGCATGATGCAAAGGCTTATGCTGTGTGTATACATCGCTACGAATTCTCCCGGGGTACCTTGCAAACACAGTCCGG

>TRINITY_DN74387_c0_g3_i1 YP_003800000.1 proline-alanine-rich protein [Spissistilus festinus virus 1] from the St_IT2 library

TGCAGCACGTAACCTCACCTCTTTCATCTTATCATCTTGGGATTACTACGACAACAGCTACATGTACGCCAAGCTGTGGTACTATGTGCTGCTAAATGACCTAATTGCATCTGTGGACGGGACACCCACTGCAATTGCCTTTTCTCAAAACCCAGTTGTACCCAACTGGATCAACATTGCCGACCAGAATTTGGATGTCAATGCAGTCCCAAATGCCATCTTACGGCGAGATATCATCTTAATTGATAATAAAGACATTAATGCCACCAACGCAACTGATTTACAGTTTGTTTATTGGCTCACCAACTCGGGGATGCGCTATGACGGACCACAGGGGTCTCCAACTCCACATGCCATTTATGTGGAATGGTCTAGCATCCCTATCACCGTTATATCACACGTCCAAGCCCCCAGTGCACCCCAGGCCGCCATCATCACCTCTCCCAGCTTGATTGCGATCATACATAGATTAGCTAGTAGCAGGGTAGAGTGGACATCTGCGCTGAAGGGGCTTTATTTAGCCATGGATATATTGGGG
