# Supplementary material for: New Viral Sequences Identified in the Flavescence Dorée Phytoplasma Vector Scaphoideus titanus
Source: Viruses. 2020 Mar 6;12(3):287. doi: 10.3390/v12030287 (PMC7150801; doi:10.3390/v12030287)
Supplement: Supplementary file 1 [file viruses-12-00287-s001.zip › Supplementary _Materials/Supplementary_materialS3.doc]

Supplementary material S3: List of primers used in this work.

| **primer name** | **primer sequence (5'-3')** | **Target virus** | **product size**  **(bp)** | **Annealing T**  **(°C)** | **amplification on** |
| --- | --- | --- | --- | --- | --- |
| StEU_toti-likef | CAGGAACGACACACAGAACC | Scaphoideus titanus toti-like virus 1 | 271 | 65 | cDNA |
| StEU_toti-liker | TCAAACATCACTTGTACGCCC |  |  |  |  |
| StEU_ifla1f | CGCCCTTCGCAAAAGAACTC | Scaphoideus titanus iflavirus 1 | 214 | 64 | cDNA |
| StEU_ifla1r | AGAAGTACCCATAGTGGCAC |  |  |  |  |
| StUSA_sobemo-like1_RdRpf | GTCTGTCAACGGCGATGAAC | Scaphoideus titanus sobemo-like virus 1 | 405 | 62 | cDNA |
| StUSA_sobemo-like1_RdRpr | GTGAACACGAGTAGATCAAG |  |  |  |  |
| StUSA_sobemo-like1_hypf | CAGCACCGTCAGGCATCTC | Scaphoideus titanus sobemo-like virus 1 | 257 | 64 | cDNA |
| StUSA_sobemo-like1_hypr | GAAGGCACGACAAGGAAATC |  |  |  |  |
| StUSA_sobemo-like2_RdRpf | ACAGTAGTACAACAGGTCCC | Scaphoideus titanus sobemo-like virus 2 | 424 | 64 | cDNA |
| StUSA_sobemo-like2_RdRpr | CCTTTCCTATCGCCAGTGTC |  |  |  |  |
| StUSA_sobemo-like2_hypf | GCGAGAGCAGTAAAATGCAC | Scaphoideus titanus sobemo-like virus 2 | 435 | 64 | cDNA |
| StUSA_sobemo-like2_hypr | CTTAGTTGGGGGAACATCTG |  |  |  |  |
| StUSA_permutof | GCCGACACGATGGATTGCTC | Scaphoideus titanus permutotetra-like virus 1 | 362 | 68 | cDNA |
| StUSA_permutor | GAACACTTGCCAGCCTTCTG |  |  |  |  |
| StUSA_ifla1f | GTAGTTAGGGAGCATAAGAAG | Scaphoideus titanus iflavirus 1 | 227 | 63 | cDNA |
| StUSA_ifla1r | GCAACGCGCACGGGGACAGG |  |  |  |  |
| StUSA_reo_RdRpf | CAATGGCGCAACGGAAGTG | Scaphoideus titanus reovirus 1 | 421 | 64 | cDNA |
| StUSA_reo_RdRpr | CGTCAGGATCAAGGTCATCA |  |  |  |  |
| StUSA_partiti-like_RdRpf | CGGGCTCCAATGAAGATCCC | Scaphoideus titanus-associated partiti-like virus 1 | 420 | 68 | cDNA |
| StUSA_partiti-like_RdRpr | CTTTGTCGGGCACGAAACGG |  |  |  |  |
| StUSA_bunya-like_glycof | GGTGAGGAGTATTGTCGTAG | Scaphoideus titanus bunya-like virus 1 | 343 | 64 | cDNA |
| StUSA_bunya-like_glycor | CACTTTTCCATCATCCCCTG |  |  |  |  |
| StEU_EVE_mononega1f | CCACTGCTCAATCCGCTTC | Mononegavirus 1 | 361 | 60 | DNA  (EVE) |
| StEU_EVE_mononega1r | GTCTCGCTATTAAAGTAACC |  |  |  |  |
| StEU_EVE_mononega2f | GGCGAGTGTTACGGCTGTTG | Mononegavirus 2 | 513 | 62 | DNA  (EVE) |
| StEU_EVE_mononega2r | CAGAGAAACGATGCTGTACTG |  |  |  |  |
| StEU_EVE_mononega3f | AGAGAATGGTGTGTCTACC | Mononegavirus 3 | 222 | 62 | DNA  (EVE) |
| StEU_EVE_mononega3r | CCTAGATCCAACTGTGTCAGA |  |  |  |  |
| StEU_EVE_reof | ATTAGAGCCTTACGACGCAG | Reovirus | 562 | 64 | DNA  (EVE) |
| StEU_EVE_reor | ATTGACTTTGGGAAGAACCGC |  |  |  |  |
| StEU_EVE_phlebor | AGAACTTCAGATGCCGT | Phlebovirus | 222 | 60 | DNA  (EVE) |
| StEU_EVE_phlebof | TCCTTCACTATCAGTATGTC |  |  |  |  |
| StEU_EVE_toti-like1f | GGATTACTACGACAACAGCTAC | Toti-like virus | 350 | 65 | DNA  (EVE) |
| StEU_EVE_toti-like1r | CGGTGATAGGGATGCTAGAC |  |  |  |  |

Column 3 reports the specific name assigned to putative insect-infecting viruses (Scaphoideus titanus viruses) and the generic name assigned to viral sequences presumably integrated into the insect genome.

Column 6 indicates whether each primer couple was used to confirm the expression of the identified viruses by RT-PCR (cDNA) or the integration into the insect genome as EVE (DNA-EVE)
